# Efficient *ex vivo* analysis of CD4+ T-cell responses using combinatorial HLA class II tetramer staining

**DOI:** 10.1038/ncomms12614

**Published:** 2016-08-30

**Authors:** Hannes Uchtenhagen, Cliff Rims, Gabriele Blahnik, I-Ting Chow, William W. Kwok, Jane H. Buckner, Eddie A. James

**Affiliations:** 1 Benaroya Research Institute at Virginia Mason, Translational Research Program, Seattle, Washington 98101, USA; 2 Neuroimmunology Unit, Department of Neuroscience, Solna, Karolinska Institutet, Karolinska University Hospital, SE-171 76 Stockholm, Sweden; 3 Benaroya Research Institute at Virginia Mason, Diabetes Program, Seattle, Washington 98101, USA; 4 Department of Medicine, University of Washington, Seattle, Washington 98195, USA; 5 Benaroya Research Institute at Virginia Mason, Diabetes Program and Tetramer Core Laboratory, Seattle, Washington 98101, USA

## Abstract

MHC tetramers are an essential tool for characterizing antigen-specific CD4+ T cells. However, their *ex vivo* analysis is limited by the large sample requirements. Here we demonstrate a combinatorial staining approach that allows simultaneous characterization of multiple specificities to address this challenge. As proof of principle, we analyse CD4+ T-cell responses to the seasonal influenza vaccine, establishing a frequency hierarchy and examining differences in memory and activation status, lineage commitment and cytokine expression. We also observe cross-reactivity between an established epitope and recent variant and provide a means for probing T-cell receptor cross-reactivity. Using cord blood samples, we correlate the adult frequency hierarchy with the naive precursor frequencies. Last, we use our combinatorial staining approach to demonstrate that rheumatoid arthritis patients on therapy can mount effective responses to influenza vaccination. Together, these results demonstrate the utility of combinatorial tetramer staining and suggest that this approach may have broad applicability in human health and disease.

## Abstract (web summary)

Detection of antigen specificity in a blood sample is limited by low frequencies of individual clones. Here the authors develop an MHC class II combinatorial technique and apply it to immunophenotype human CD4+ T cells of multiple specificities in response to influenza vaccine in single blood samples.

## Graphical abstract

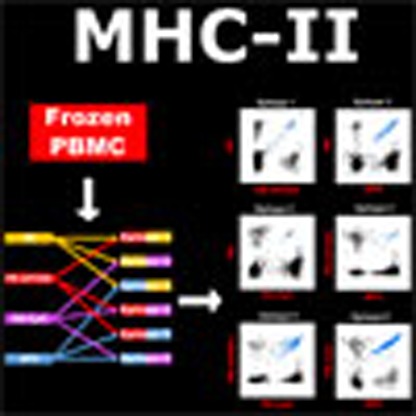

The increasing interest in translational and human immunology as well as the growing depth of clinical immunology has motivated the study of antigen-specific immune cells from inherently limited samples. Major histocompatibility complex (MHC) tetramer staining enables the characterization, quantification and sorting of defined antigen-specific T cells^1^. Protocols for the *ex vivo* tetramer staining of comparatively rare antigen-specific CD4+ T cells have provided a crucial tool for T-helper-cell analysis in basic and clinical immunology^2^,^3^,^4^. Accordingly, MHC class II tetramer staining has become an invaluable approach in immunology, enabling direct interrogation of the naturally developing T-cell repertoire, assessment of changes in T-cell responses caused by perturbations such as vaccination and disease, and providing a means of confirming the translational relevance of observations in model systems^4^,^5^,^6^,^7^,^8^,^9^,^10^,^11^,^12^.

Whereas the direct *ex vivo* analysis of antigen-specific CD8+ T cells can be accomplished with 1–2 million peripheral blood mononuclear cells (PBMCs), this generally requires 20–30 million PBMCs per epitope for antigen-specific CD4+ T cells. Therefore, sample requirements have been a particular concern and have severely limited the ability to study CD4+ T-cell responses against more than a single epitope, especially when relying on clinical sample repositories. One essential innovation addressing large cell number requirements has been the implementation of combinatorial staining strategies^13^,^14^. However, the precise enumeration and phenotypic analysis of antigen-specific CD4+ T cells remains technically difficult, mainly due to their low frequency, the comparatively weak CD4–MHC interaction and the technical challenges of recombinant MHC II production^15^,^16^. Consequently, the published combinatorial protocols are not readily applicable to MHC class II tetramers and their use has significantly lagged behind the progress made with class I tetramers. To address this need, we have developed a combinatorial tetramer staining protocol for direct enumeration and phenotypic analysis of multiple CD4+ T-cell specificities in a single staining tube, thereby significantly increasing their efficient *ex vivo* analysis.

As proof of principle for our combinatorial human leukocyte antigen (HLA, the human MHC genes) class II tetramer assay, we analyse CD4+ T cells specific for six epitopes derived from various vaccine strains of the seasonal influenza virus. CD4+ T-cell responses are an important correlate of vaccine efficacy and protection against the influenza virus^17^,^18^,^19^,^20^,^21^,^22^, and their differential boosting by the seasonal influenza vaccination provides a highly relevant setting for the parallel characterization of multiple specificities e*x vivo*. For our study, we select epitopes derived from influenza type A strains that were included in either the 2014–2015 northern hemisphere influenza vaccine or in previous vaccines. We simultaneously examine the number, memory and activation phenotype, lineage commitment and cytokine expression of the influenza-specific T-cell responses. Furthermore, we explore the relationship between the identified frequency hierarchy in adults and naive precursor frequencies by examining the frequencies of influenza-specific T cells in cord blood. Last, we investigate the influence of biologic treatments on the ability to mount effective CD4+ T-cell responses by examining influenza-specific T cells in rheumatoid arthritis (RA) patients before and after vaccination. Together, our findings provide insights about T-cell responses to the influenza vaccine and illustrate the general utility of this novel combinatorial approach to efficiently characterize CD4+ T-cell *ex vivo* from limited samples in a variety of different contexts.

## Results

### Combinatorial *ex vivo* staining analysis of six specificities

HLA class II *ex vivo* tetramer staining protocols are well established in our laboratory and have enabled the routine detection and characterization of rare CD4+ T cells from human PBMCs^2^,^3^,^4^,^11^,^23^. However, using existing techniques, a single characterization of the six epitopes selected for this study would require up to 150 million PBMCs per subject (∼150 cc of blood) at each time point—a number that is generally not reconcilable with sample repositories. Therefore, we aimed to develop a novel combinatorial tetramer staining protocol for the parallel detection of multiple CD4+ T-cell specificities from single staining tubes of 20–30 million PBMCs.

**Figure 1:**
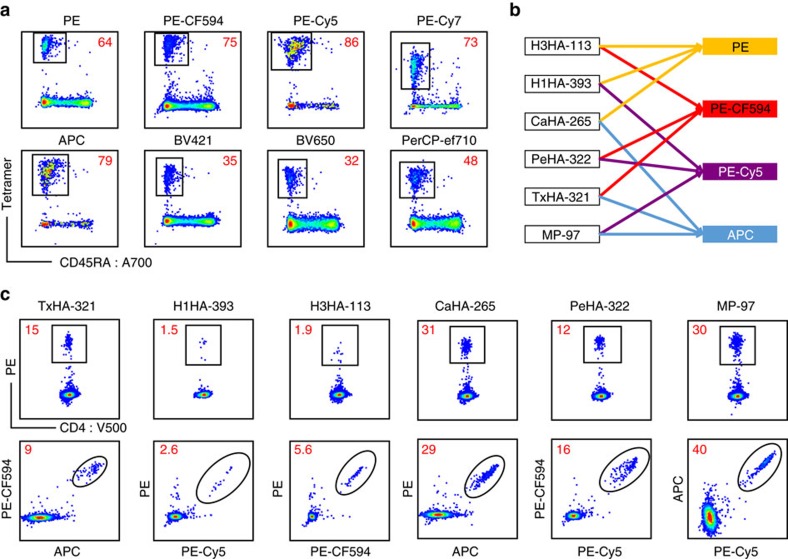
Multi-colour and combinatorial HLA class II staining. Validation of additional fluorophores for HLA II tetramer staining and the combinatorial staining protocol from frozen PBMCs from healthy donors. Tetramer+ frequencies were calculated as cells per million CD4+ and are displayed in red in each flow cytometry plot. Examples are representative of three independent experiments and the gating strategies for all tetramer staining are shown in Supplementary Fig. 3. (**a**) *Ex vivo* tetramer staining with MP-97 tetramers conjugated with eight different fluorophores performed in individual staining tubes reveals PE-Cy7, BV421, BV650 and PerCP-ef710 to be suboptimal. (**b**) Schematic representation of the combinatorial staining approach in which each of the six epitopes is encoded by a unique combination of two fluorophores. (**c**) The six epitopes detected by either single tetramer staining in individual tubes or combinatorial staining in a single tube. Summary statistics of this comparison are shown in Supplementary Fig. 4.

As previous experience indicated that the choice of tetramer fluorophore might have a substantial impact on staining performance^24^, we conducted side-by-side testing of eight promising streptavidin–fluorophore conjugates to identify the most useful labels for combinatorial staining. Traditionally, tetramer enrichment is dependent on the binding of magnetic beads to either PE or APC^2^,^3^,^4^. To facilitate simultaneous enrichment of tetramer+ cells with any label of interest, we added a c-Myc tag to the c-terminal end of the HLA alpha chain, which allows enrichment of tetramer-bound cells independent of their fluorophore^25^ (Supplementary Fig. 1). We next compared the efficacy of tetramers conjugated with eight different fluorophores by staining for the dominantly recognized MP-97 epitope. PBMCs from a healthy control subject were stained with tetramers labelled with APC, PE, PE-CF594, PE-Cy5, PE-Cy7, BV421, BV650 or PerCP-ef710. Of the tested fluorophores, we found that only PE, PE-CF594, PE-Cy5 and APC consistently delivered comparable *ex vivo* tetramer results, judging based on both the frequency and median fluorescence intensity (MFI) of the enriched tetramer-positive T cells (Fig. 1a). In contrast, labelling with BV421 and BV650 resulted in ∼50% fewer events, labelling with PE-Cy7 tetramers led to dimmer staining with increased background, and labelling with PerCP-ef710 conjugated tetramers led to dimmer staining and fewer events. These differences were apparently not due to the different method of enrichment, as either anti-PE or anti-Myc enrichment of PE-labelled cells gave essentially identical results (Supplementary Fig. 1). Rather, as we had previously observed for tetramer labelling of *in vitro* expanded T cells, conjugation of class II tetramers from streptavidin labelled with different fluorophores appears to generate reagents that label T cells with different efficacies, apparently favoring the large protein fluorophores (PE, PE tandems and APC). Using the four most effective fluorophores, we next developed a combinatorial staining protocol for the parallel detection of six epitopes in a single staining tube (Fig. 1b). The protocol was validated using the influenza epitopes listed in Table 1, demonstrating that all fluorophore combinations yielded effective and reliable double staining (Fig. 1c). Importantly, these tetramers could be used together in the same tube without significant bias or spectral overlap. The combinatorial protocol also allowed a stringent gating strategy to be applied that assured the removal of events that were positive for more than two fluorophores as well as accounting for background due to insufficient tetramer staining (Supplementary Fig. 2). The full gating scheme for the combinatorial data is outlined in Supplementary Fig. 3. Overall, our results demonstrated that combinatorial tetramer staining could successfully detect multiple epitopes with results that are comparable to single staining across a wide range of T-cell frequencies (Supplementary Fig. 4).

### Characterizing CD4+ T cells before and after vaccination

With the ability to detect the six selected epitopes of interest in a single staining tube, we designed and executed a proof-of-principle study to characterize changes in influenza-specific CD4+ T-cell responses elicited by the 2014–2015 northern hemisphere seasonal influenza vaccine. This vaccine included the A/Texas/50/2012 (H3N2) and A/California/7/2009 (H1N1) influenza A strains (along with one or two influenza B strains). Because epitopes had not been previously defined for A/Texas/50/2012, tetramer-guided epitope mapping^26^ was performed to identify DRB1*04:01 restricted haemagglutinin (HA) T-cell epitopes (Supplementary Fig. 5). All epitopes included in the study are listed in Table 1. All of the selected epitopes except the H3N2 HA epitope PeHA-322 were present in the 2014–2015 vaccine strains. PeHA-322 was replaced by TxHA-321 in 2012 and these epitopes differ at two residues, including an asparagine (N) to serine (S) change at the TCR-contacting position 326 (N326S), which is very likely to have an impact on T-cell recognition^27^,^28^. The other variant residue 323 (just outside of the binding cleft at p-1) appears as an R in all recent H3N2 vaccine strains, but a R323K variant of this sequence has been consistently used in tetramer and crystallography studies; this change in sequence has no apparent effect on tetramer binding^29^. Therefore, to maintain consistency with prior tetramer studies we elected to use the R323K variant. PBMCs were collected from six HLA-DRB1*04:01-positive subjects before vaccination and after receiving the 2014–2015 seasonal northern hemisphere influenza vaccine (Table 2). The time of blood draw after vaccination ranged from 14 to 21 days with a mean of 17 days. Extensive surface phenotyping was performed together with combinatorial tetramer staining to measure the frequency, memory status (CD45RA and CCR7), chemokine receptor expression (CXCR3, CCR4, CCR6 and CXCR5) as well as activation status (CD38) of these influenza-specific T cells.

**Figure 2:**
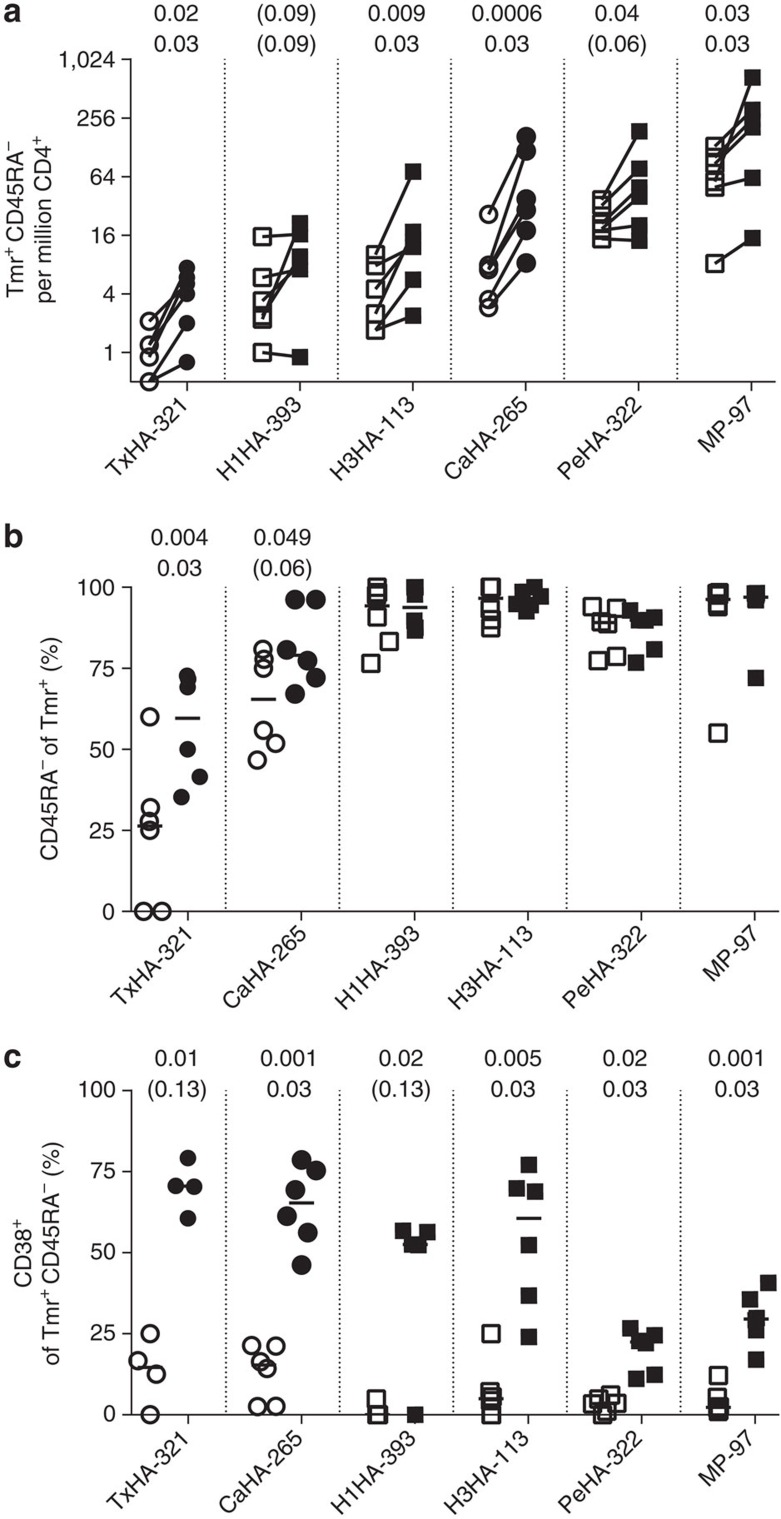
CD4+ T-cell response to the seasonal influenza vaccination. T-cell frequencies and vaccine response for the six subjects and the four conserved (squares) and two recently introduced epitopes (circles) obtained with the combinatorial staining protocol from frozen PBMCs. (**a**) Memory (CD45RA^−^) frequencies before (open symbols) and after (closed symbols) vaccination revealing significant increased median frequencies for four of the epitopes and trending increases for the other two. (**b**) Analysis of memory frequencies reveals that only the recently introduced TxHA-321 and CaHA-265 epitopes have median memory levels significantly below 90% before vaccination and display significant increases with vaccination (Supplementary Fig. 6a). (**c**) CD38 levels for the different antigens were low before vaccination and showed significant increases with vaccination for all epitopes except the now-absent PeHA-322. CD38 levels were high after vaccination except for MP-97 and PeHA-322, which had significantly lower levels after vaccination (Supplementary Fig. 6b). Statistical significances are calculated from paired *t*-tests (upper values) and Wilcoxon matched-pairs signed rank test (lower vaues) after log-transforming the data (for panel **a**). *P* values ≥0.05 are indicated by parenthesis.

Since all subjects should have an existing memory repertoire of influenza-specific T cells, we first quantified the frequency of memory (CD45RA^−^) tetramer-positive cells before and after vaccination. Overall, the frequencies were found to be of the magnitude expected for antigen-specific CD4+ T cells in peripheral blood^3^,^4^,^5^,^30^ ranging from several hundred cells per million CD4+ (∼1 in 5,000) for the conserved MP-97 epitope to less than one per million for TxHA-321. Following vaccination, increased frequencies were observed for each of the epitopes in the expected range of a two to eightfold increase (Fig. 2a)^30^,^31^, with statistically significant increases for TxHA-321, CaHA-265, H3HA-113 and MP-97. Despite this variable expansion, the response hierarchy observed before vaccination appeared largely unchanged by seasonal vaccination (Fig. 2a).

### Memory status and phenotype of influenza-specific T cells

Analysis of memory status via CD45RA expression revealed that most influenza-specific CD4+ T cells were of the memory phenotype (Fig. 2b) as expected given that all epitopes had been included in the vaccine since at least 2012. Interestingly, TxHA-321 and CaHA-265, the two epitopes introduced more recently in the seasonal vaccine, still displayed a significantly lower proportion of memory cells before vaccination. These frequencies remained below those of the other four epitopes despite an increase after vaccination (Supplementary Fig. 6a), indicating a slow conversion of the naive repertoire. Separation of effector and central responses via the expression of CCR7 did not reveal additional differences between the responses (Supplementary Fig. 7). The activation status of the antigen-specific CD4+ T cells was assessed by measuring surface levels of CD38, a marker of recent T-cell activation^32^,^33^. As expected, CD38 levels were low for the influenza-specific cells before vaccination and displayed a significant increase after vaccination for all epitopes except PeHA-322, which was absent in the vaccine strains (Fig. 2c). After vaccination, T cells specific for PeHA-322 and unexpectedly also T cells specific for the universally conserved MP-97 epitope were found to have significantly lower levels of CD38 compared with the other epitopes (Supplementary Fig. 6b).

### Chemokine receptor expression and T-helper lineage analysis

**Figure 3:**
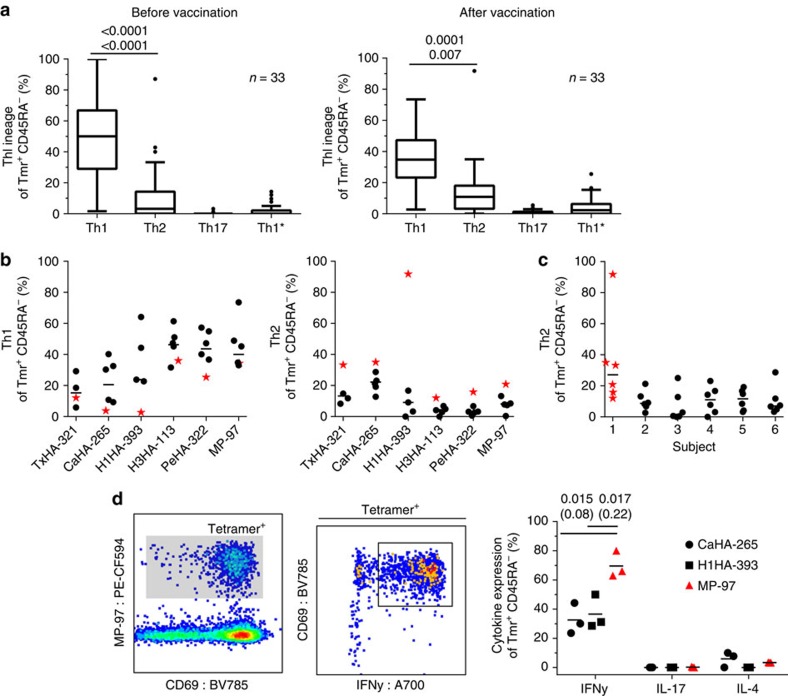
T-helper lineage analysis of the antigen-specific CD4+ T cells. Phenotypic lineage analysis of the tetramer-positive memory (CD45RA^−^) cells from the combinatorial staining. (**a**) Combined quantification of the lineage composition of tetramer-specific T cells against all epitopes and from all subjects demonstrates the predominance of Th1 cells at either time point. Data are shown as box plots with tukey bars to indicate the distribution of the data. (**b**) Analysis of Th1 and Th2 frequencies for the different epitopes after vaccination. T cells specific to TxHA-321 and CaHA-265 appear to trend towards lower and higher levels of Th1 and Th2, respectively, while cells from subject 1 (red star) consistently displayed increased frequencies of Th2 cells. (**c**) Quantification of the Th2 frequencies for all epitopes combined for the differed subjects. (**d**) Intracellular expression of IFN-γ, IL-4 and IL-17 in cells from cells stained with single tetramers for CaHA-265, H1HA-393 and MP-97 for three of the study subjects after vaccination following short stimulation with phorbol myristate acetate/ionomycin. Tetramer+ cells were gated (dark gate) and cytokine expression quantified, demonstrating dominance of IFN-γ expression and significantly higher expression in MP-97 positive cells (red triangle) compared to the other populations. Selected statistical significances are displayed from one-way analysis of variance with Holm–Sidak correction (upper values) and Kruskal–Wallis test with Dunn's correction (lower values) after comparison of all columns. *P* values ≥0.05 are indicated by parenthesis.

We also analysed the surface expression of common chemokine receptors and assigned the cells to lineages based on established patterns of chemokine receptor expression^34^,^35^: Th1 (CXCR3+, CCR4− and CCR6−); Th2 (CXCR3−, CCR4+ and CCR6−); Th17 (CXCR3−, CCR4+ and CCR6+); and Th1* (CXCR3+, CCR4− and CCR6+). As expected, the largest proportion of influenza-specific CD4+ T cells were of Th1 lineage both before and after vaccination^30^,^31^,^36^,^37^,^38^ (Fig. 3a. Quantification of individual surface receptors (including CXCR5) is shown in Supplementary Fig. 8). Analysing the lineage composition of the individual epitope responses revealed moderate variation in that epitopes TxHA-321 and CaHA-265 were characterized by lower memory frequencies (Fig. 2b) and trended towards having lower Th1 frequencies (Fig. 3b). Interestingly, we noticed that a single subject (subject 1) consistently displayed significantly increased Th2 frequencies compared with the other study participants (Fig. 3c).

The ability to functionally characterize CD4+ T cells directly *ex vivo* could substantially improve the assignment of helper lineages of antigen-specific T-cell responses as they are shaped and maintained *in vivo*^39^. We therefore devised a protocol in which individual tetramer staining and enrichment directly precedes activation with phorbol myristate acetate/ionomycin and intracellular cytokine staining. For three of the study subjects (subject 1 and two other representative subjects), T cells specific to CaHA-265, CaHA-393 and MP-97 were tested for intracellular interferon-γ (IFN-γ), interleukin (IL)-4 and IL-17 after vaccination to demonstrate the feasibility of *ex vivo* functional characterization. We observed robust upregulation of CD69 and IFN-γ following activation with only modest impact on tetramer staining (Fig. 3c). As expected (and in line with chemokine receptor expression) influenza-specific CD4+ T cells expressed mainly IFN-γ, along with very low but detectable levels of IL-4 and IL-17 (Fig. 3c). Overall, a higher frequency of IFN-γ+ cells was observed for MP-97-specific T cells, matching the higher levels of Th1 lineage assignment based on surface chemokine receptor expression (Fig. 3b).

### Naive precursor frequencies match hierarchies after exposure

**Figure 4:**
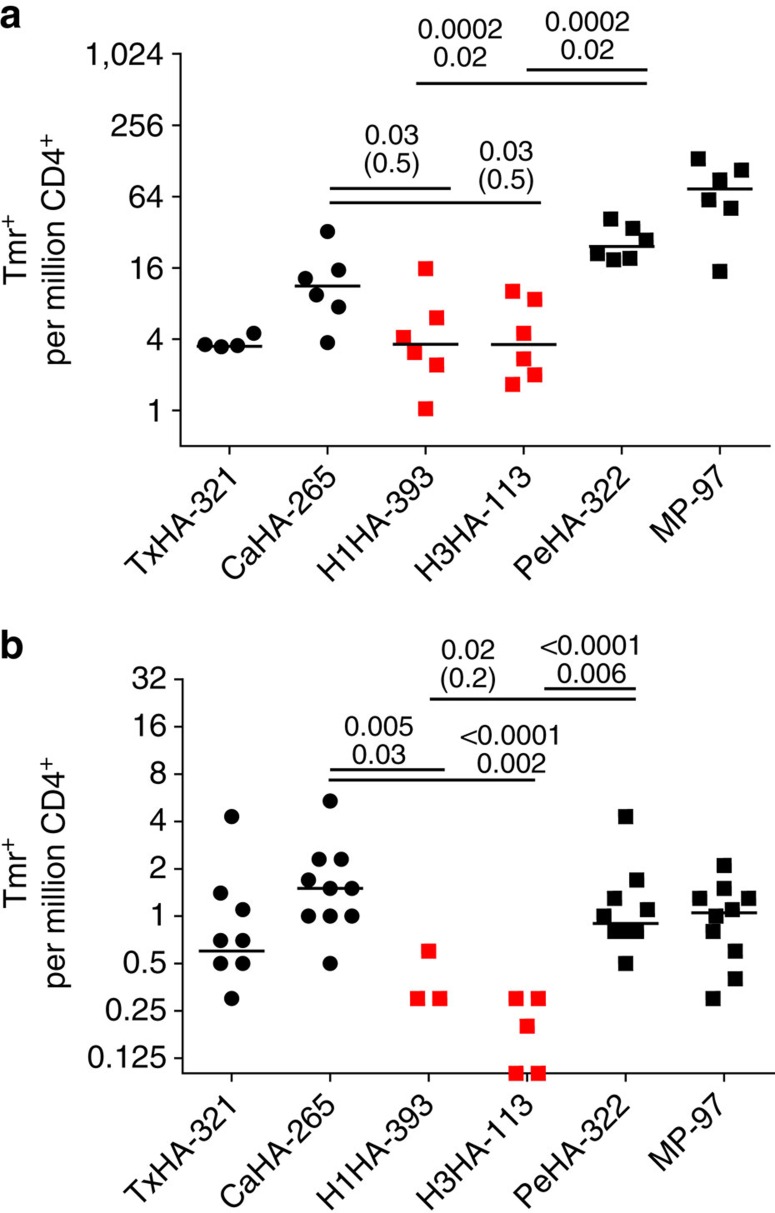
Naive precursor frequencies in cord blood. Frequency of total tetramer+ T cells determined before vaccination in the study subjects and in frozen PBMCs derived from cord blood of 10 healthy controls with the combinatorial tetramer staining. The recently introduced (circles) epitopes are followed by the conserved targets (squares) and responses to the conserved epitopes H1HA-393 and H3HA-113 are highlighted in red. (**a**) T-cell frequencies in adults before the 2014 influenza vaccination. H1HA-393 and H3H1-113 are recognized significantly less than the equally conserved H3 epitope PeHA-322 and even trending lower than the recently introduced H1 epitope CaHA-265. (**b**) The T-cell frequencies found in cord blood mirror the hierarchy observed in adults with the cells specific for H1HA-393 and H3HA-113 barely detectable. Statistical significances have been calculated with one-way analysis of variance with Holm–Sidak correction (upper values) and Kruskal–Wallis test with Dunn's correction (lower values) after log-transforming the data and comparison of the conserved epitopes H1HA-393 and H3HA-113 (red) with the newly introduced CaHA-265 and conserved PeHA-322. *P* values ≥0.05 are indicated by parenthesis.

The combined analysis of responses to all six epitopes in each subject revealed a largely consistent epitope hierarchy with the conserved epitope MP-97 followed by the different HA epitopes, PeHA-322, CaHA-265, H3HA-113 and H1HA-393 with the most recently introduced variant epitope TxHA-321 at the bottom (Fig. 2a). Interestingly, in this hierarchy, T cells against two of the conserved epitopes, H1HA-393 and H3HA-113, were found at relatively low frequencies when compared not only with the conserved H3 PeHA-322 epitope but even in comparison with the relatively recently introduced H1 CaHA-265 epitope (Fig. 4a). We speculated that these differences could be related to frequency differences in the naive precursor repertoire. Unfortunately, it is essentially impossible to query the true naive precursor repertoire in children or adults due to frequent and very early exposure to the virus and the seasonal vaccine. However, neonates can be safely considered to lack exposure to the virus. Therefore, we applied the developed combinatorial staining protocol on cord blood samples from 10 healthy HLA-DRB1*04:01+ donors as a means of interrogating the naive repertoire. As expected, antigen-specific T-cell frequencies were low, in the range of 0.1–4 per million CD4 (Fig. 4b)^5^,^40^, and the cells were completely naive. As in the post-vaccination samples, significant differences existed between different epitopes and these were largely consistent between different subjects, demonstrating that shared factors shape T-cell frequencies in the naive repertoire. Notably, we found that H1HA-393 and H3HA-113 were present at the lowest frequencies among the epitopes tested and significantly below PeHA-322 and CaHA-265, suggesting that these specificities are under-represented in antigen-experienced adults because of their scarcity in the naive repertoire.

### Analysis of cross-reactivity between TxHA-321 and PeHA-322

**Figure 5:**
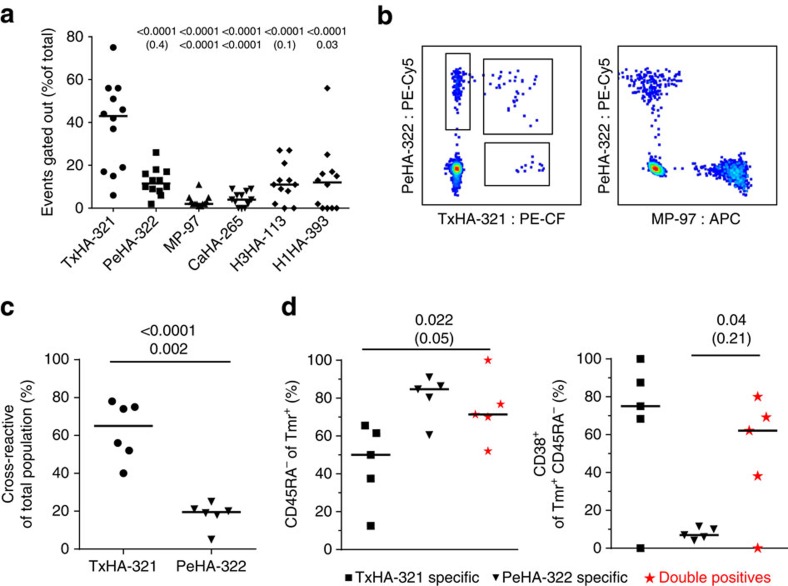
Cross-reactivity between PeHA-322 and TxHA-321. Assessment of cross-reactivity in the combinatorial data and then specifically between PeHA-322 and TxHA-321 assessed from frozen PBMCs after vaccination samples from the six study subjects. (**a**) The percentage of events gated out during the gating procedure from the combinatorial data for each of the epitopes strongly indicates cross-reactivity in the TxHA-321-specific population. (**b**) PBMCs from all subjects before and after vaccination were stained with tetramers for TxHA-321 in PE-CF594, PeHA-322 in Pe-Cy5 and MP-97 in APC to analyse cross-reactivity as well as an unrelated specificity in PE to remove events stained non-specifically. Representative plot of CD4+ cells demonstrating the staining of single- and double-positive events between PeHA-322 and TxHA-321 as well as the absence of cross-reactivity between the other epitopes. (**c**) Quantification of the degree of cross-reactivity within each of the two tetramer populations. While on average, 50% of the TxHA–321–specific T-cell population is also stained by PeHA-322 only a small fraction of the total PeHA-322 population displays cross-reactivity. (**d**) The memory and activation phenotype of the cross-reactive population (red star) was compared with the single-specific population after vaccination. The cross-reactive cells feature a combination of memory-high and CD38-high that is not found in either of the single-specific populations. Selected statistical significances are displayed from one-way analysis of variance with Holm–Sidak correction (upper values) and Kruskal–Wallis test with Dunn's correction (lower values) after comparison of all columns. *P* values ≥0.05 are indicated by parenthesis. Group in **c**, where compared with *t*-test and Mann–Whitney test.

Among the sequences included in our study, the TxHA-321 and PeHA-322 epitopes are homologous, differing by the N326S modification at a predicted TCR-contacting residue^27^,^28^, whereas other pairs of epitopes were essentially non-homologous. This scenario raises the possibility that TxHA-321 and PeHA-322 could be targeted by cross-reactive T cells. Counting only cells that are positive for exactly two tetramer labels is a key component of combinatorial gating approaches to prevent over-estimation of T-cell frequencies through non-specific tetramer binding and spectral overlap. However, a consequence of this gating requirement is that any cells that cross-recognize more than one of the epitopes in the panel are lost from the analysis. Conversely, comparing the observed frequency of tetramer-positive events for each epitope before and after the removal of events positive for additional tetramer colours provides a means of detecting cross-reactivity between pairs of epitopes. Applying this strategy revealed that on average ∼40% of events in the TxHA-321 tetramer gate were positive for at least one other tetramer colour and thus excluded from the final analysis (Fig. 5a). This indicates either an unusual degree of non-specific binding or significant cross-reactivity with another epitope. Further analysis demonstrated that these losses were largely accounted for by additional positivity for colours corresponding to PeHA-322 and (to a lesser degree) MP-97 tetramers (Supplementary Fig. 9). None of the other epitopes showed elevated signs of cross-reactivity and the observed events were widely distributed amongst the other tetramer colours, suggesting non-specific staining.

To confirm the apparent cross-reactivity between PeHA-322 and TxHA-321 and rule out non-specific binding, we performed follow-up staining experiments with PBMCs from all six subjects before and after vaccination. This staining panel used single tetramer colours to label TxHA-321 (PE-CF), PeHA-322 (PE-Cy5), MP-97 (APC) and an unrelated control tetramer (PE). Cross-reactivity was found between TxHA-321 and PeHA-322 that was highly specific and completely absent from PeHA-322 and MP-97 (Fig. 5c). The cross-reactive population constituted only a fraction of the more frequent PeHA-322-specific response but made up on average half of the total number of T cells that recognized the newly introduced TxHA-321 epitope (Fig. 5c). Notably, T cells specific to PeHA-322 versus TxHA-321 differed significantly with respect to their activation and memory status (Fig. 2b,c). When analysing the phenotype of cross-reactive T cells, it became clear that they share characteristics of the two specific populations, displaying a high memory frequency similar to that of PeHA-322 reactive cells (consistent with expansion in response to previous influenza strains) together with strongly upregulated CD38 after vaccination (indicating activation by the 2014 vaccine) as seen for the TxHA-321-specific cells (Fig. 5c).

### Influenza vaccine responses of RA patients

**Figure 6:**
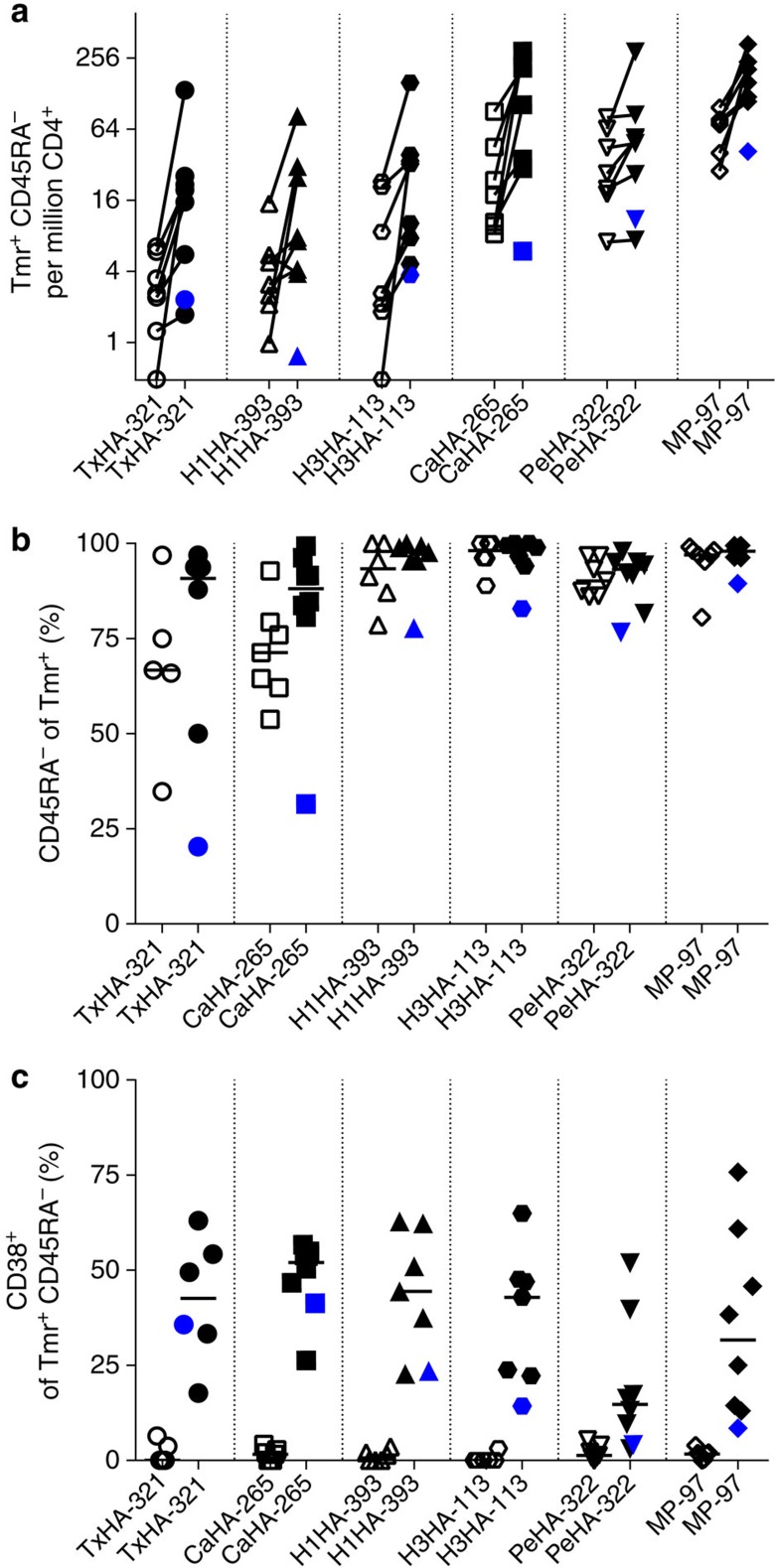
Response to vaccination in RA patients. (**a**) Memory (CD45RA^−^) T-cell frequencies, (**b**) memory and (**c**) activation phenotypes have been analysed from frozen PBMCs of RA patients receiving the 2015 seasonal vaccine analogous to data presented in Fig. 2. Subject 8, which was on CLTA4-Ig treatment at the time of vaccination is highlighted in dark blue.

The reduced cell requirements of the combinatorial class II tetramer protocol provides a means to analyse the response to influenza vaccination in other areas of clinical relevance. Specifically, we now had a convenient tool to comprehensively analyse how the CD4 T-cell response to influenza vaccination is shaped by RA and its treatments. Epidemiological data indicate that RA patients have an increased risk of hospitalization after infection^41^. For influenza vaccination in particular, previous studies using HA inhibition antibodies as a surrogate measure of protection have suggested that RA patients on biologics (most notably CLTA4-Ig and B-cell depletion) have decreased responses to the influenza vaccine^42^. We recruited eight patients that were sampled before and after the 2015 seasonal influenza vaccine and covered a spectrum of common immune-modifying treatments (Table 3). In spite of the immunomodulatory and immunosuppressive treatments that these patients received, we observed no overall significant differences in the CD4+ T-cell responses elicited by the 2015 seasonal vaccine between RA patients on biologics and healthy controls (Fig. 6; Supplementary Fig. 10). Analysing specifically the subject receiving a CTLA4-Ig, it appears that the drug may block memory conversion (Fig. 6b), particularly for the Tx-HA-321 and CaHA-265 epitopes, which still have a sizable naive repertoire. However, additional studies would be needed to confirm this observation.

## Discussion

Tetramer staining provides essentially the only practical means of directly analysing antigen-specific T cells *ex vivo* without activation or other manipulation. This technique has enabled fundamental discoveries in basic and clinical immunology^1^,^4^,^5^,^6^,^7^,^8^,^9^,^10^,^11^,^12^ and holds great promise for studying the *in vivo* plasticity, differentiation and cross-reactivity of antigen-specific CD4+ T cells—research areas that have received increasing attention over the past years^35^,^43^,^44^,^45^,^46^. We have expanded the range of fluorophores that can be used for HLA class II tetramer staining and developed a novel combinatorial staining protocol for the parallel *ex vivo* staining and analysis of six epitopes in a single staining tube. This enables the study of antigen-specific CD4+ T-cell responses from limited samples as well as truly comprehensive epitope characterization when large sample volumes are available. As reported in its application for MHC class I tetramers^13^,^14^, the combinatorial gating strategy also allowed a highly effective removal of non-specific events, flagging of suboptimal double staining and gating of truly specific cells.

Not all of the tested fluorophores performed equally well when compared with PE, despite the fact that some labels (such as Qdot and Brilliant Violet colours) provided a relatively high staining MFI. This aligns with previous observations that certain fluorophores were not suitable for effective tetramer staining of *in vitro* expanded T cells^24^. The exact reason for these differences is unclear but, based on our experience, we think it is possible that tetramers labelled with large fluorophores such as PE have more favourable performance because they exhibit an increased tendency to aggregate into higher-order multimers, facilitating improved labelling of lower-affinity T cells. In this case, other MHC multimer backbones (such as dextramers or dodecamers) could enable the use of additional fluorophores that have not performed well in our studies^47^,^48^,^49^. For example, there has been some success staining CD4+ T cells with fluorescein isothiocyanate (FITC)-labelled dextramers even though this label is ineffective for tetramers^50^. Using the four optimal fluorophores, we found that all of the possible staining pairs performed well in combination, which should obviate the need for extensive optimization before adoption of our protocol in other laboratories. In addition, the developed staining protocol allows for an extensive characterization of surface phenotypes, effectively combining screening and analysis without the need of additional sample, as exemplified here in the combined analysis of recent T-cell activation and T-cell helper lineages for all six epitopes.

We chose seasonal influenza vaccination for a proof-of-principle study to demonstrate the feasibility and utility of the developed combinatorial staining protocol. Cross-reactive CD4+ T-cell recognition of conserved influenza epitopes particularly in the M1 matrix protein (MP) and nuclear protein is well established and believed to provide protection against heterologous challenges^17^,^18^,^19^,^20^,^51^,^52^,^53^,^54^. However, whereas neutralization and cross-recognition of the less conserved but essential neutralization target HA by flu-specific antibodies is studied extensively and with increasing sophistication^55^,^56^,^57^,^58^,^59^, the influences of the seasonal heterologous vaccination on the frequency, function and phenotype of CD4+ T-cell responses are less well-understood^30^. Attempting to address this, the six influenza epitopes selected for our study can be broadly divided into three categories: (i) three epitopes are conserved and essentially present in any seasonal influenza vaccine (H3HA-113, H1CaHA-393 and MP-97); (ii) one epitope has been present in the vaccine for many years but is absent since 2012 (PeHA-322); and (iii) two epitopes have been introduced only over the last few years with the pandemic H1N1 strain in 2009 (CaHA-265) and the H3N2 Victoria strain in 2012 (TxHA-321, which replaced PeHA-322). Examining the frequency of T cells that recognize these six epitopes, we found a response hierarchy that was largely consistent among the study participants, suggesting shared factors driving epitope dominance in these HLA-DRB1*04:01-positive individuals. This hierarchy could not be entirely explained by the time each epitope had been included in the vaccine, but revealed that individuals maintain frequent memory T cells specific to the now-absent PeHA-322 epitope and that the relatively recently introduced CaHA-265 epitope is targeted more frequently than two of the conserved epitopes.

To characterize further the factors shaping frequency hierarchies, we performed the first systematic analysis of the antigen-specific naive precursor frequencies from human cord blood. Using cord blood is important, as it is essentially impossible to otherwise characterize naive precursors to influenza antigens and homeostatic expansion as well as cross-reactivity is likely to influence the repertoire even in truly unexposed adults^60^,^61^. As in adults there were significant and consistent differences between the epitopes and the two hierarchies strongly resembled each other. These results provide an explanation for the comparatively low recognition of the conserved H1HA-393 and H3HA-113 epitopes, and the prominence of the more recently introduced CaHA-265 epitope. Furthermore, they confirm previous observations with mouse CD4+ T cells^4^ as well as with human CD4+ T cells from unexposed adults^5^ regarding the influence of naive precursor frequencies on the resulting hierarchy of memory T cells. Interestingly, these results differ from findings in human CD8+ T cells, where consistent hierarchies existed in cord blood but these did not correspond to those found in exposed adults^6^. Overall, these results strongly suggest that the frequency hierarchy of CD4+ T cells specific to seasonal influenza vaccination and viral exposure is at least partially determined at the stage of repertoire generation and thymic selection.

As expected for responses against the influenza virus, the antigen-specific T cells were of memory phenotype, upregulated the activation marker CD38 after vaccination^30^,^33^ and were of the Th1 lineage when characterized by surface receptor expression^30^,^31^,^36^,^37^,^38^. Detailed analysis demonstrated that the two recently introduced epitopes (TxHA-321 and CaHA-265) stood out through exhibiting significantly decreased memory frequencies and, conversely, responses to both MP-97 and PeHA-322 were distinguished by lower levels of CD38 after vaccination, suggesting that these T cells may not be fully activated by the vaccine. This is not surprising for PeHA-322, which should not be present in the vaccine; however, frequencies of T cells against this epitope still trended higher after vaccination and were significantly increased for MP-97. It remains unclear whether the lack of CD38 upregulation is caused by differences in antigen presentation and availability or related to bystander activation, as has been recently suggested^62^,^63^,^64^, but the approach presented here would provide a convenient means to address this in subsequent studies.

In this study, we leveraged the combined staining of six epitopes in a single tube together with the specific gating strategy to search for signs of cross-reactivity between the specificities tested. This led to the discovery of cross-reactivity between the close variants PeHA-322 and TxHA-321. The cross-reactive T cells that recognized both PeHA-322 and TxHA-321 were found to display shared features from both populations. Notably, a large proportion of PeHA-322-specific cells did not recognize the newer TxHA-321 epitope with high affinity, suggesting that the N326S mutation may act as an escape variant that renders the relatively high pre-existing memory T-cell pool less effective against newly emerged strains that carried the new sequence variant. Apart from potentially explaining the slight differences in T-cell frequency observed between combinatorial and single staining, the observation of cross-reactivity also indicates that the reported frequencies against the TxHA-321 epitope are low estimates. However, accounting for these does not appear to fundamentally change the observed frequency hierarchy. Similarly, the memory and activation phenotype results need be to be interpreted in light of the analysis done on the different TxHA-321-specific populations and their different propensity for memory and CD38 expression. The observed results suggest that inspection of data obtained from combinatorial tetramer staining for excessive losses of events during gating should be a routine step that can have important implications for data interpretation. The topic of cross-reactivity within the TCR repertoire could have wider significance for antigen-specific T-cell research, as TCR with a broad tolerance for changes in the MHC/peptide-binding interface could be of particular importance for the development of broadly protective vaccines or could be a distinguishing feature of pathogenic autoreactive T cells. In scenarios where large numbers of epitopes are visualized in parallel, unexpectedly high levels of cross-reactivity between different epitopes could be observed *de novo* using the approach that we have outlined here.

In summary, we present here a novel combinatorial HLA class II tetramer staining protocol that facilitates deep analysis of multiple CD4+ T-cell specificities from limited samples. This strategy enables efficient characterization of the frequency and phenotype of epitope-specific T cells and facilitates the identification of cross-reactivity. Our results strongly suggest that the naive precursors in cord blood play an important role in shaping later hierarchies and they provide a platform for further studies of the truly naive antigen-specific T-cell repertoire. The developed protocol can yield important mechanistic insights about T-helper-cell responses in the setting of vaccination, and provides an opportunity to routinely link the characterization of T-helper-cell response with serology, as well as in other clinical research settings such as the study of responses to pathogens, allergens and self-antigens.

## Methods

### Sampling and HLA typing of vaccinated human subjects

Subjects and cord blood samples included in the study were positive for HLA-DRB1*04:01 and enroled in the Benaroya Research Institute healthy control registry or rheumatic disease registry with informed consent and samples donated under studies approved by the Benaroya Research Institute Institutional Review Board. Six subjects scheduled to receive the 2014–2015 influenza vaccine shot (Fluzone, Sanofi Pasteur or Fluvirin, Novartis; Table 2) were sampled on average 3 days (ranging from 0 to 12 days) before as well as on average 17 days (ranging from 14 to 21 days) after vaccination. These subjects had an average age of 53 years of age (ranging from 42 to 67 years). In addition, three healthy control subjects that had previously received the 2014–2015 influenza vaccine were sampled for tetramer-guided epitope mapping. Subjects receiving the 2015–2016 vaccine were collected on average 15-day post vaccine (range 11–20) and on average 62 years old (range 39–70) as well as on a range of medications (Table 3). For each subject, HLA-DR was typed using Dynal Unitray SSP Kits according to the manufacturer's instructions (Invitrogen, Carlsbad, CA, USA), or by PCR amplification with sequence-specific primers.

### Generation of the c-Myc DRB1*0401 expression construct

The c-Myc tag (EQKLISEEDL) was fused to the C terminus of the extracellular DR-alpha chain basic leucine zipper protein following a single GS linker. The extracellular domain of the DR-beta chain was fused with an acidic leucine zipper and BirA recognition sequence. Both constructs were subcloned into a metallothionein-inducible vector pMT/V5-His A (Thermo Fisher Scientific, MA, USA). Sequencing-verified plasmids were transfected into Drosophila S2 cells via Cellfectin II (Thermo Fisher Scientific) following the manufacturer's recommendation. Fourty-eight hours post transfection, transfection mixtures were removed and replaced with selection medium containing 10% fetal bovine serum medium and 1 mg ml^−1^ G418 (Thermo Fisher Scientific). Transfected cells were selected for 2 weeks and cells that produced the highest levels of soluble proteins were expanded subsequently for large-scale production^65^.

### Antibodies and tetramers used in the study

Fluorescent antibodies used in this study were purchased from BD Biosciences (San Jose, CA, USA) and BioLegend (San Diego, CA, USA). A list of all antibodies used in this study can be found in Supplementary Table 1. Conjugated streptavidins were purchased from BD Bioscience (PE-CF594, PE-Cy7 and PE-Cy5), BioLegend (BV421, APC, PE and BV650) or eBioscience (PerCP-ef710; San Diego, CA, USA). Myc-tagged or -untagged HLA class II monomer reagents were generated essentially as previously described^65^ by the BRI tetramer core. In brief, transfected S2 cells were expanded to a 2 l volume in spinner flasks (Bellco, Vineland NJ) and induced for 5 days with 1 mM copper sulfate, adding 2 μg ml^−1^ biotin to ensure efficient protein biotinlyation. Supernatants were separated from intact cells by centrifugation (11,000*g*), separated from debris using a 0.2 μm filter (ThermoFisher, Waltham, MA, USA) and then affinity purified using L243 coupled with CNBr-Activated Sepharose 4B (GE Healthcare, Pittsburgh, PA, USA). Class II protein was eluted at pH 11.5, equilibrated using pH 4.0 Tris buffer and exchanged into a pH 6.0 storage buffer (0.2 M sodium phosphate). As needed, class II monomers were loaded with individual peptides by incubating for 72 h at 37 °C in the presence of 2.5 mg ml^−1^ *n*-octyl-β-D-glucopyranoside (Sigma, St Louis, MO, USA). Tetramers were formed by individually incubating class II molecules with labelled streptavidin for 6–18 h at room temperature at a molar ratio of 8:1, except for APC-conjugated tetramers, which were conjugated at 8:0.75 (75% of the calculated streptavidin-APC amount).

### Peptides and tetramer-guided epitope mapping

A peptide library of HA from the H3N2 Texas strain (A/Texas/50/2012; GenBank accession AGL07159.1) was purchased with 70 peptides each 20 amino acids long (except the last peptide, which was 16 amino acids long) with a 12 amino-acid overlap (Mimotopes, Clayton, Australia). HLA-DRB1*04:01-restricted epitopes from the HA sequence of the A/Texas/50/2012 strain were identified by stimulating PBMCs from three healthy subjects first with pools of five overlapping peptides and tested for tetramer staining at day 14. Tetramer-positive pools were then stained individually with the five relevant tetramers and epitopes with clear staining in at least two donors considered positive^26^. Positive epitopes were selected and further validated through *ex vivo* tetramer staining for antigen-specific CD45RA^−^ memory T cells.

### HLA class II tetramer enrichment and analysis

*Ex vivo* tetramer staining and enrichment was based on previously published protocols^30^. A total of 30–40 million PBMCs were thawed and rested for 1 h in a tissue culture incubator. Tetramer staining was performed in 14 ml round-bottom polystyrene tubes (Corning) in 200 μl T-cell culture medium, following Dasatinib treatment for 10 min at 37 °C (ref. 66). A measure of 4 μl of each tetramer was added followed by incubation at room temperature for 90 min. Cells were then incubated with 40 μl of anti-PE and anti-APC magnetic beads or 10 μl of anti-c-Myc magnetic beads (Miltenyi Biotec, San Diego, CA, USA) and enriched on a mass spectrometry-sized magnetic column according to the manufacturer's instructions. Before enrichment, a 1/100th cell fraction was set aside for antibody staining (‘Pre'). Enriched cells and ‘Pre' samples were surface stained for 20 min at 25 °C with CD38-BUV395, CD4-V500 and CD45RA-A700 (BD Bioscience), and CD14-FITC, CD19-FITC, AnnexinV-FITC, CXCR3-BV421, CCR4-BV605, CCR6-BV785, CCR7-APC-Cy7, CXCR5-PE-Cy7 (Biolegend).

Each sample was collected to completion on a BD LSR Fortessa flow cytometer. Data were analysed using FloJo v10.1 and GraphPad Prism 6.05. The frequency (*F*) of epitope-specific T cells per million CD4+ T cells was calculated as follows: *F*=(1,000,000 × tetramer-positive events from enriched tube)/(100 × number of CD4+ T cells from the ‘Pre' fraction). Single T cells were gated CD4+/CD14− CD19−AnnexinV− and a double-negative population was gated for each of the six possible two-colour combination of tetramer fluorophores. These served as the parent populations from which the six tetramer double-positive T-cell populations were then gated and analysed for surface receptor expression (for example, APC+PE-Cy5 double positive was gated based on the double-negative population in the PE+PE−CF594 plot). All relevant gating strategies are displayed in Supplementary Fig. 3. For the analysis of memory frequencies before and after vaccination on a logarithmic scale, frequencies of zero were converted to 0.5, a conservative estimate of the limit of detection^5^. T-cell lineage was assigned on CD45RA^−^ memory cells as follows: Th1 (CXCR3+, CCR4− and CCR6−); Th2 (CXCR3−, CCR4+ and CCR6−); Th17 (CXCR3−, CCR4+ and CCR6+); Th1* (CXCR3+, CCR4− and CCR6+)^34^,^35^.

### *Ex vivo* tetramer Intracellular cytokine staining

Tetramer staining and enrichment was performed as described above, except that T cells were rested overnight in the incubator in 5 ml aliquots in 50 ml conical tubes at a concentration of 2 million PBMCs per ml. Tetramer-enriched cells and ‘Pre-enriched' cells were incubated in a 96-well U-bottom plate in the presence of 50 ng μl^−1^ phorbol myristate acetate and 1 μg ml^−1^ ionomycin for 3.5 h with 5 μg ml^−1^ Brefeldin A (BioLegend) added after 30 min. Surface staining was then performed with CD4-BV510 (BD Bioscience) and CD14-FITC, CD19-FITC, AnnexinV-FITC and CD45RA-PE-Cy7 (BioLegend). Cells were then fixed and permeabilized using the Bioscience intracellular staining and permeabilization buffer set according to the manufacturer's instructions. Intracellular staining was performed with IFN-γ-A700, IL-4-A647, IL-14-APC-Cy7 and CD69-BV785 (BioLegend).

### Statistical analysis

Because it is inherently challenging to confirm the normal distribution and lack of skewing for data sets with small sample sizes, we have elected to employ both parametric and nonparametric tests for all statistical comparisons. In each case, *P* values derived from both parametrical and nonparametrical tests for statistical significance are annotated in each figure. Comparisons before and after vaccination in Figs 2 and 4 have been performed using paired *t*-tests and the Wilcoxon nonparametric test. Comparisons of data across epitopes or subsets (Figs 3 and 5a,d) were performed with one-way analysis of variance and the Kruskal–Wallis test including multiple comparisons of either all possible comparisons or *a priori* specified comparisons as indicated in the respective figure legends. Individual columns in Fig. 5c were compared using a *T*-test and the Mann–Whitney *U*-test. All displayed *P* values have been corrected for multiple testing whenever appropriate using Holm–Sidak (parametrical tests) or Dunn (nonparametrical tests) corrections. All *P* values are two-tailed and values ≥0.05 are indicated by parenthesis. The tetramer frequency data displayed in Fig. 2a, Fig. 4 and Supplementary Fig. 10 were log-transformed before statistical analysis.

### Data availability

The data that support the findings of this study are available from the corresponding author on request.

## Additional information

**How to cite this article:** Uchtenhagen, H. *et al*. Efficient *ex vivo* analysis of CD4+ T-cell responses using combinatorial HLA class II tetramer staining. *Nat. Commun.* 7:12614 doi: 10.1038/ncomms12614 (2016).

## Supplementary Material

Supplementary Information Supplementary Figures 1-10 and Supplementary Table 1

Peer review file

## Figures and Tables

**Table 1:** Influenza epitopes used in this study.

**Name**	**Protein**	**Sequence**	**Vaccine strain**	**Reference**
MP-97	MP	97-VKLYRKLKREITFHGAKEIS	*Universally conserved*	51
H3HA-113	HA	113-CYPYDVPDYASLRSLVASSG	A/Texas/50/2012 H3N2 (conserved in H3 HA)	This study
H1HA-393	HA	393-TNKVNSVIEKMNTQFTAVGK	A/California/7/2009 H1N1 (conserved in H1 HA)	30
CaHA-265	HA	265-LVVPRYAFAMERNAGSGIII	A/California/7/2009 H1N1 (introduced in 2010)	30
TxHA-321	HA	321-CP**R**YVKQ***S***TLKLATGMRNVP*	A/Texas/50/2012 H3N2 (introduced in 2012)	This study
PeHA-322	HA	322-P**K**YVKQ***N***TLKLAT*	— (absent since 2012)	65

^*^The sequence shared between TxHA-321 and PEHA-322 is underlined and differing epitopes are shown in bold with the N326S mutation in italics.

**Table 2:** Healthy subjects included in this study.

**Subject**	**Time of draw (days)**	**Date of vaccination**	**Type of vaccine**	**Gender**	**Age (years)**	**HLA**		
						**HLA-A**	**HLA DRB1**	**HLA DQB1**
1	0	10/8/2014	Fluzone	Female	66	02, Unknown	04:01, 11:03	03:01, 03:02
	15							
2	−12	10/13/2014	Fluvirin	Male	42	02, Unknown	04:01, 11:02	03:01, 03:02
	14							
3	−7	10/9/2014	Fluzone	Male	44	02, Unknown	04:01, 10:01	05:01, 03:02
	14							
4	0	10/8/2014	Fluzone	Female	45	not 02, not 02	04:01, 03	02, 03:02
	19							
5	0	10/14/2014	Fluvirin	Female	56	02, Unknown	04:01, 01	Unknown, Unknown
	21							
6	−1	9/23/2014	Fluvirin	Female	67	02, Unknown	04:01, 11:01	03, 03
	20							

HLA, human leukocyte antigen.

**Table 3:** Rheumatoid arthritis subjects included in this study.

**Subject**	**Date of vaccination**	**Gender**	**Age**	**HLA DRB1**	**Medication type**
1	10/15/2015	Female	69	04:01, 03	DMARD†
2	10/26/2015	Female	39	04:01, unknown	TNF blockade‡
3	10/12/2015	Female	52	01, 04:01	DMARD†
4	10/5/2015	Male	47	04:01, 01	TNF blockade§
5	11/6/2015	Female	69	04:01, 04:01	B-cell depletion||
6	10/20/2015	Female	57	04:01, unknown	Jak inhibitor¶
7	11/24/2015	Male	48	03, 04:01	TNF blockade#
8*	9/22/2015	Female	63	04:01, 04:08	CTLA4-Ig**

HLA, human leukocyte antigen.

^*^Only a post vaccine sample was available for subject 8.

^†^Disease modifying anti-rheumatic drug, methotrexate.

^‡^Adalimumab.

^§^Etanercept.

^||^Rituximab.

^¶^Tofacitinib.

^#^Simponi.

^**^Abatacept.
