# Supplementary material for: Efficient ex vivo analysis of CD4+ T-cell responses using combinatorial HLA class II tetramer staining
Source: Nat Commun. 2016 Aug 30;7:12614. doi: 10.1038/ncomms12614 (PMC5013714; doi:10.1038/ncomms12614)
Supplement: Supplementary Information — Supplementary Figures 1-10 and Supplementary Table 1 [file ncomms12614-s1.pdf]

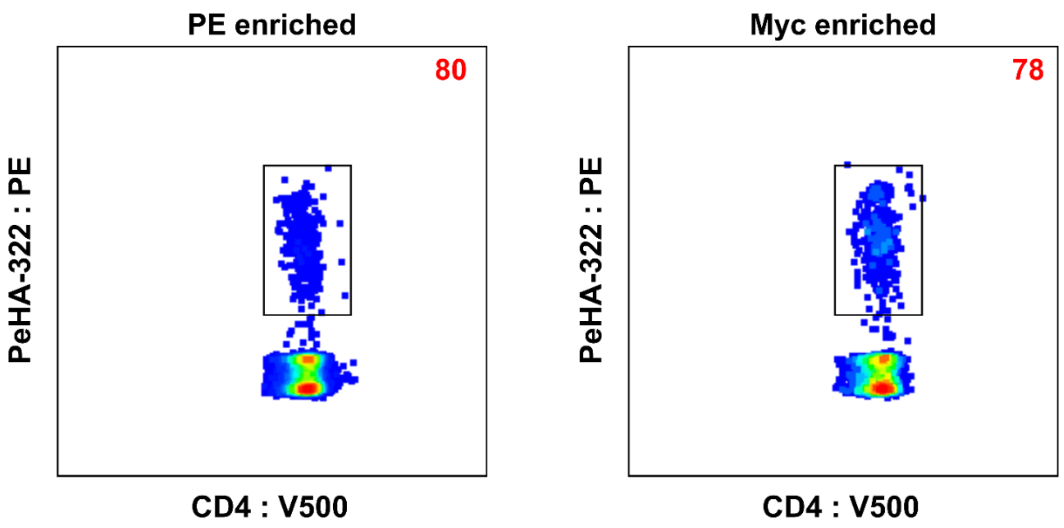


**Supplementary Fig. 1:** ***Ex vivo*** **tetramer enrichment with anti-c-Myc beads**

Representative example of comparative *ex vivo* tetramer enrichment performed in three independent experiments with either conventional anti-PE bead or anti-c-Myc bead-enriched tetramer positive cells from frozen PBMC of healthy controls. Tetramer positive events are calculated per million CD4+ and displayed in each plot (red numbers).

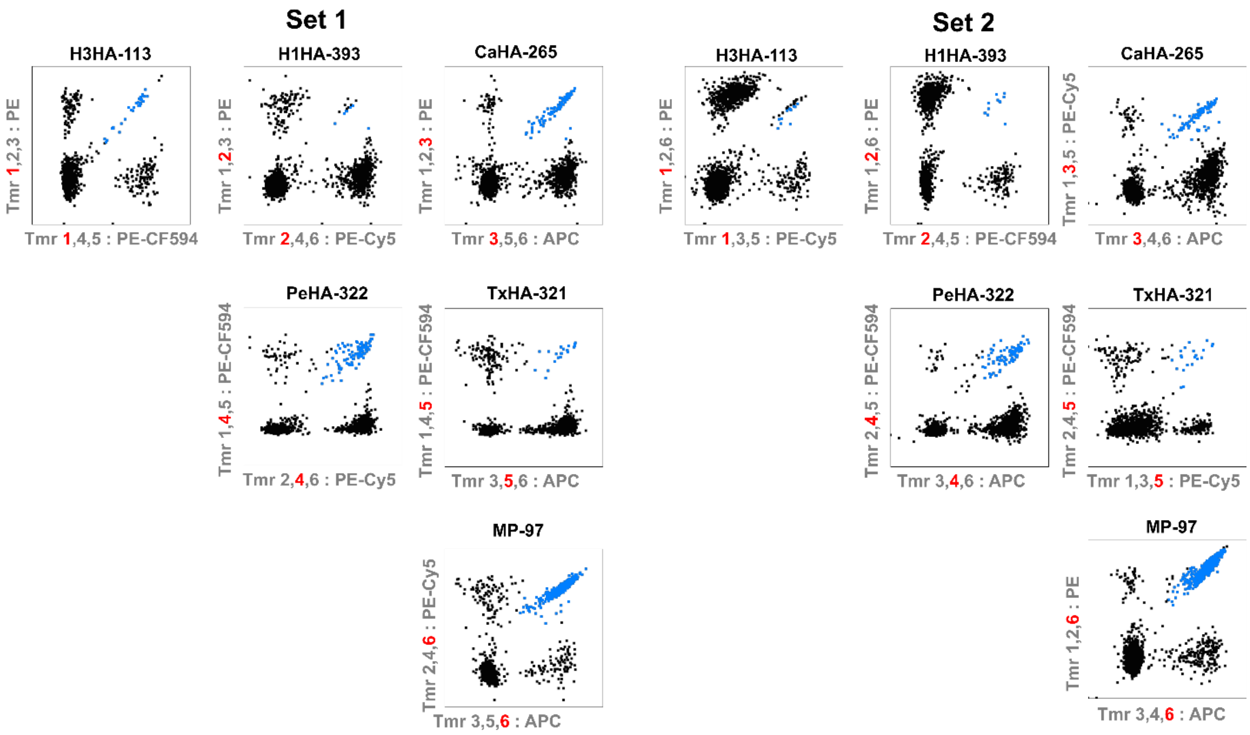


**Supplementary Fig. 2: Validation of the multiplex staining**

Representative example of two independent experiments in which fresh PBMC from a healthy control were stained with tetramers encoding the six different influenza epitopes in two different sets of color combinations. The epitope number encoded by each of the specific Tetramer combination is shown in red. One flow cytometry plot for each of the epitopes shows the original ungated CD4+ cells in that plot (black) as well as the final population in blue. Events that were found to non-specifically stain multiple tetramers are excluded from the final population and can be seen in black.

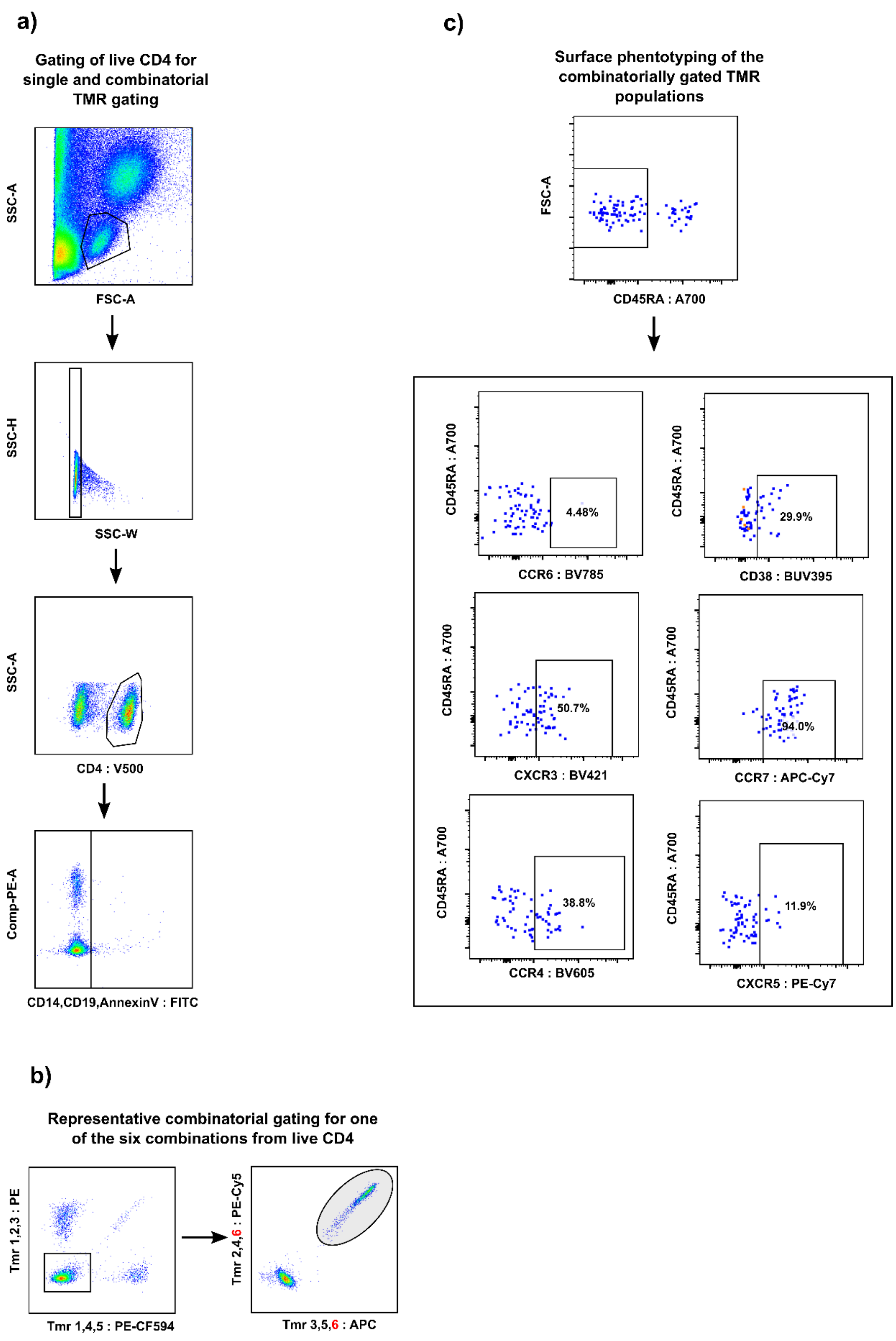


**Supplementary Fig. 3: Gating strategies**

(**a**) Gating scheme of live CD4 T-cells. **(b)** Representative example of the combinatorial tetramer gating. **(c)** Gating scheme for the memory, lineage and activation marker phenotyping of gated tetramer populations.

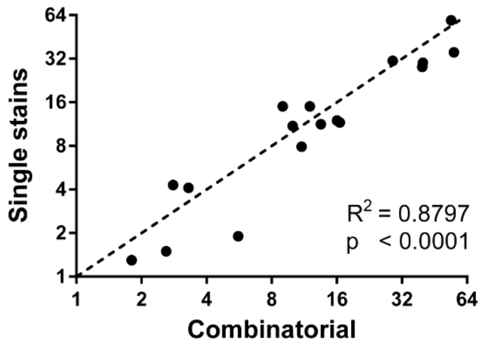


**Supplementary Fig. 4: Correlation between the combinatorial and single staining protocols**

Correlation of the comparison of single *vs.* combinatorial staining with linear regression statistics calculated from log-transformed data and performed with fresh and frozen PBMC from healthy donors.

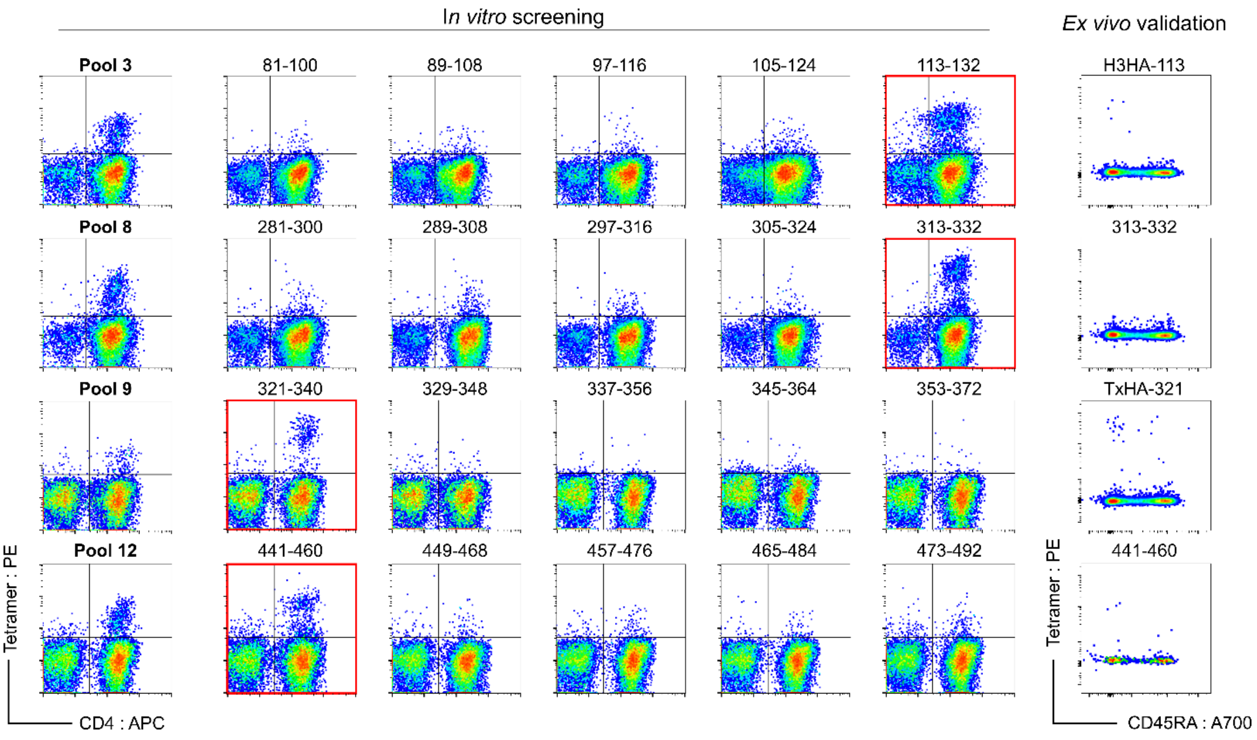


**Supplementary Fig. 5: Mapping of CD4+ epitopes of the A/Texas/50/2012 influenza strain.**

Three healthy subjects where drawn after having received the 2014 seasonal vaccine and PBMC stimulated with pools of 5 overlapping 20-mer HA peptides and a representative example is displayed. A total of 7 peptides elicited positive responses and the 4 strongest candidates were further validated by *ex vivo* tetramer staining (Fig. 1). Three of these peptides were found to be recognized by CD45RA- CD4+ T cells *ex vivo* confirming their processing and presentation *in vivo*. Of these, the apparently universally conserved H3HA-113 epitope as well as a novel epitope TxHA-321 were selected for this study.

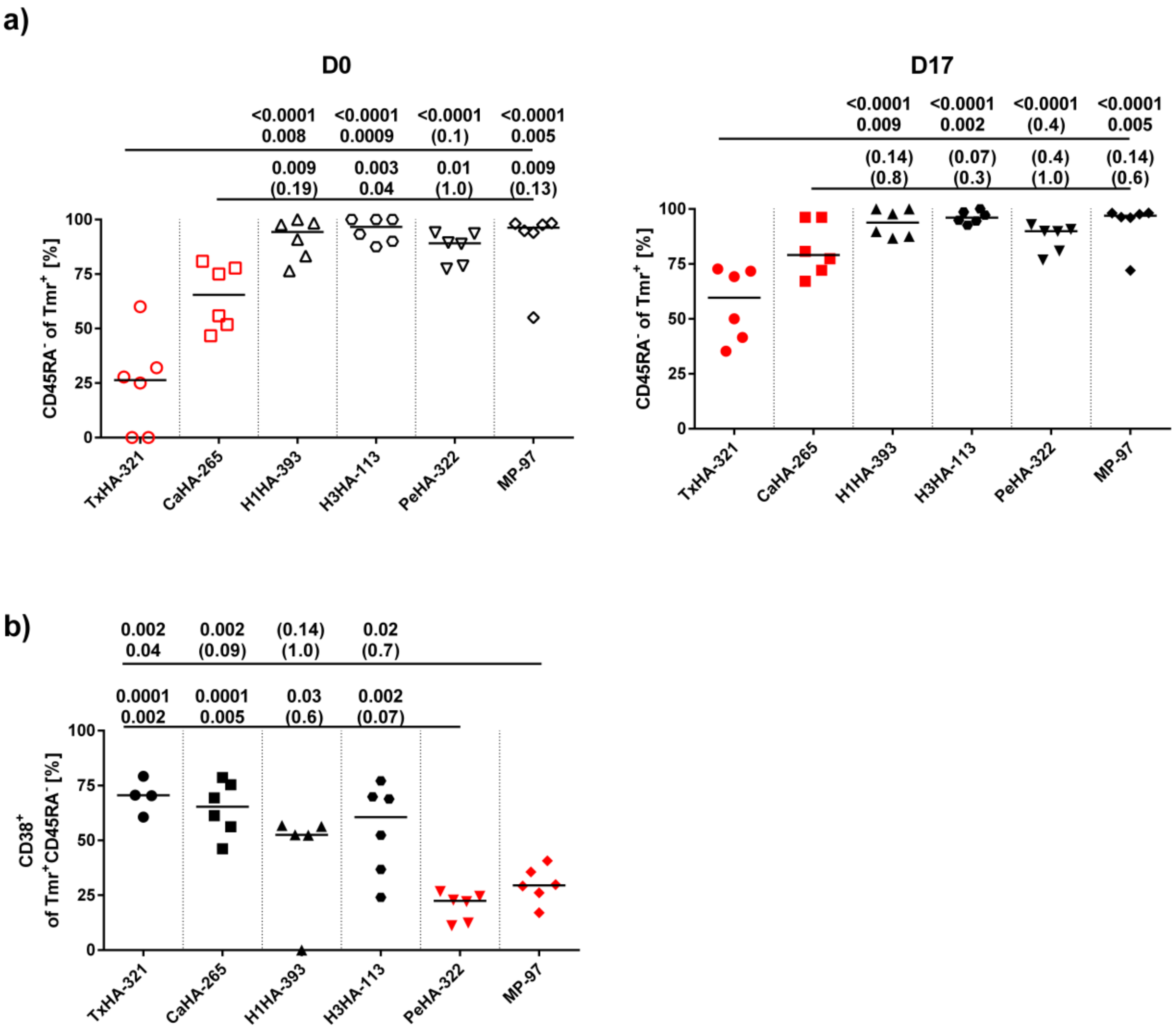


**Supplementary Fig. 6: Differences between epitopes in memory and CD38 phenotype**

**(a)** At D0 (left) T-cells specific for the newly introduced TxHA-321 and CaHA-265 (red) have lower memory frequencies compared to the other specificities (p values for comparisons with CaHA-265 and TxHA-321 are shown on the lower and upper line, respectively). At day D17 (right) responses against TxHA-321 remain significantly lower in memory frequency, while those against CaHA-265 are relatively similar to the other specificities. **(b)** CD38+ frequencies after vaccination for cells specific to PeHA-322 and MP-97, which failed to significantly upregulate the marker (red), are significantly lower than the other specificities (p values for comparisons with MP-97 and PeHA-322 are shown on the upper and lower line, respectively). Statistical significances have been calculated with one-way ANOVA with Holm-Sidak correction (upper value) and Kruskal-Wallis test with Dunn's correction (lower value) after comparison of the epitopes highlighted in red with the others. p values greater or equal 0.05 are indicated by parenthesis.

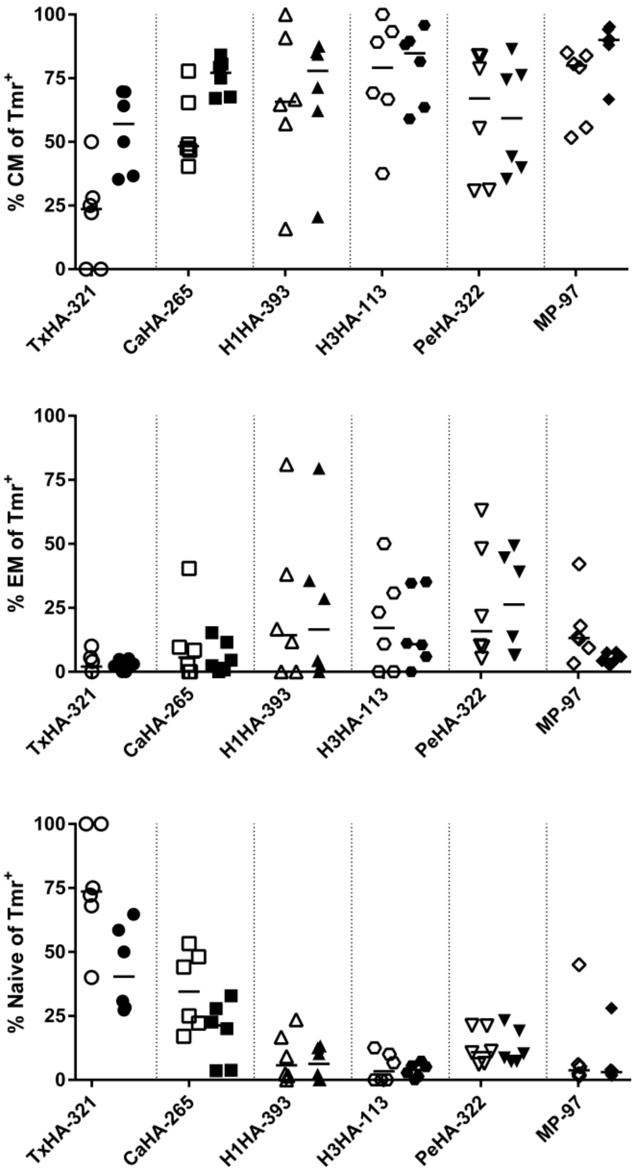


**Supplementary Fig. 7: Detailed analysis of memory phenotypes**

Quantification of the memory phenotypes of the antigen-specific CD4+ T-cells based on the expression of CD45RA and CCR7. Central memory (CM) defined as CD45RA-CCR7+, Effector memory (EM) as CD45RA-CCR7+, Naïve as CD45RA+CCR7+. Terminal effector memory cells (TEMRA) CD45RA+CCR7- were either absent or observed at <2.5% of the antigen-specific cells.

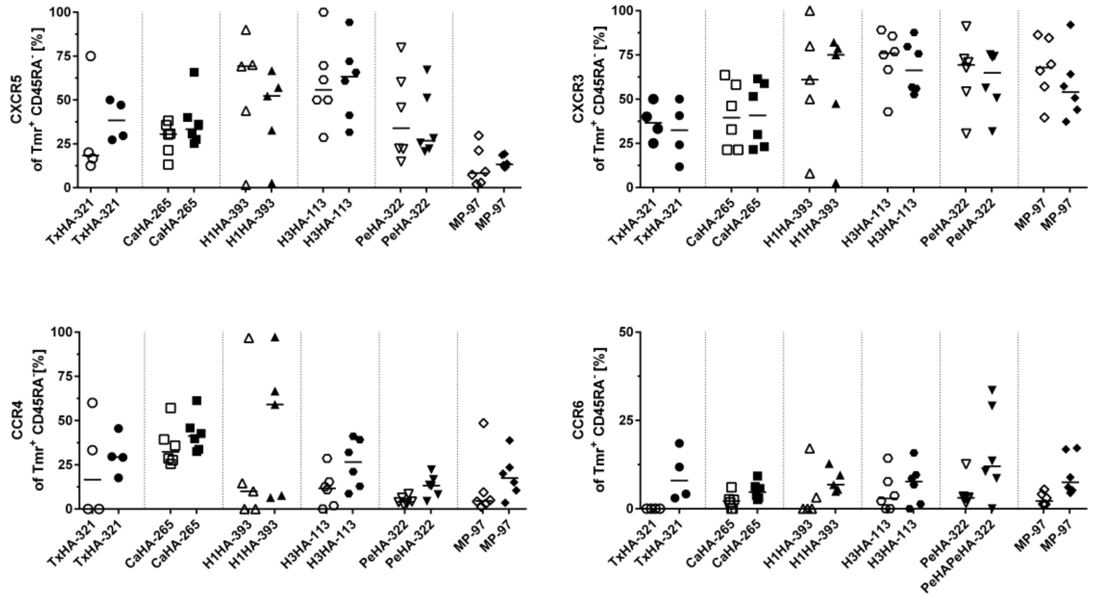


**Supplementary Fig. 8: Individual surface marker quantification**

Tetramer+ memory (CD45RA-) events were analyzed individually for the expression of CXCR5, CXCR3, CCR4 and CCR6. In line with the frequencies of Th1 and Th2 cells, TxHA-321 and CaHA-265 are trending towards and higher and lower frequencies of CXCR3 and CCR4 expression, respectively.

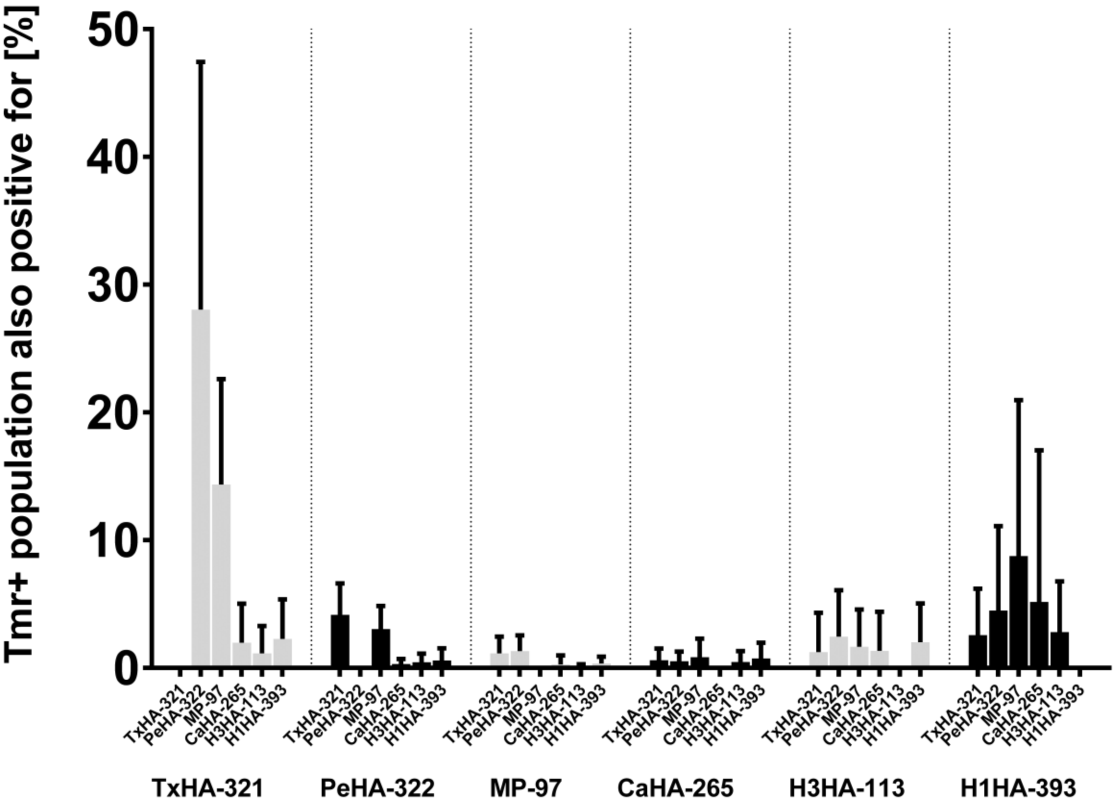


**Supplementary Fig. 9: Use of combinatorial staining to detect cross-reactivity between the targeted epitopes**

Without applying the combinatorial gating strategy we directly gated the populations specific for the six different epitopes. In order to understand why events where gated out by the combinatorial gating (Fig. 5) the additional tetramer positivity of each of the six populations was calculated in percent of total.

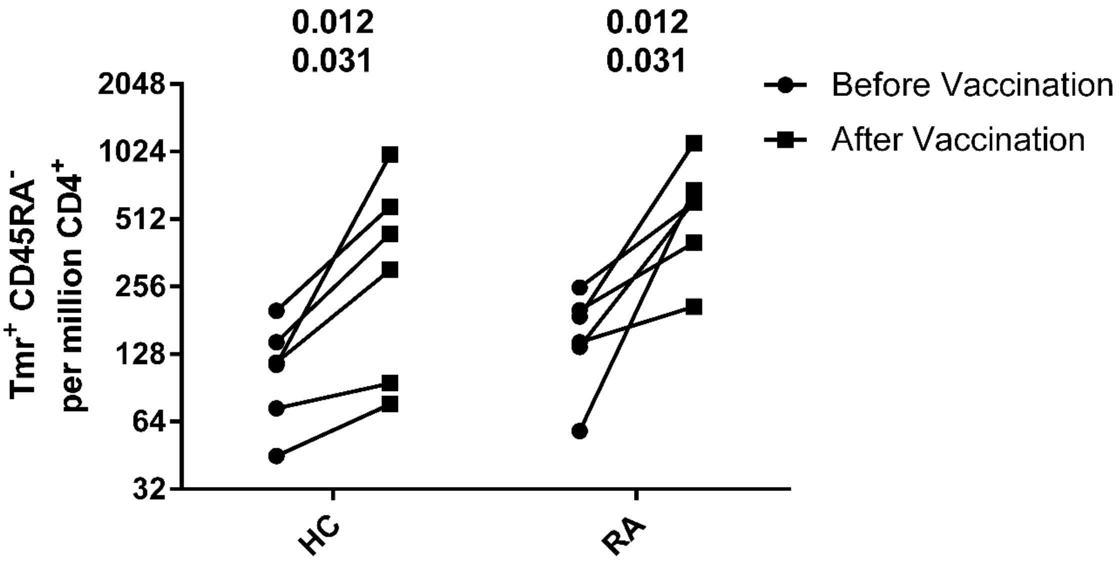


**Supplementary Fig. 10: Frequency boost of the epitope-specific CD4+ T-cells by the seasonal vaccine in healthy controls and RA patients**

Additive frequencies of the memory CD4+ T-cells specific to the six different epitopes before and after vaccination indicating a lack of substantial differences between the healthy controls and RA patients. Statistical significances in were calculated with pairing of samples before and after vaccination using the paired t-test (upper p values) and Mann-Whitney test (lower p values).

**Supplementary Table 1: Antibodies used in this study**

| Antibody | Volume (per 100 ul) | Experiment | Company | Catalogue # |
|---|---|---|---|---|
| CD14 – FITC | 2.5 | All ex vivo tmr | Biolegend | 325604 |
| CD19 – FITC | 2.5 | All ex vivo tmr | Biolegend | 302206 |
| AnnexinV – FITC | 2.5 | All ex vivo tmr | Biolegend | 640906 |
| CD38 – BUV395 | 5 | Combinatorial | BD | 563811 |
| CXCR3 – BV421 | 2.5 | Combinatorial | Biolegend | 353716 |
| CD4 – V500 | 2.5 | All ex vivo tmr except ICS | BD | 560768 |
| CCR4 – BV605 | 1.5 | Combinatorial | Biolegend | 359418 |
| CCR6 – BV785 | 2.5 | Combinatorial | Biolegend | 353422 |
| CD45RA – A700 | 3.5 | All ex vivo tmr except ICS | BD | 560673 |
| CCR7 – APC-C7 | 2.5 | Combinatorial | Biolegend | 353212 |
| CXCR5 – PE-Cy7 | 2 | Combinatorial | Biolegend | 356924 |
| IL-4 – A647 | 1 | ICS-tmr | Biolegend | 500712 |
| Interferon-γ – A700 | 1 | ICS-tmr | Biolegend | 502520 |
| IL-17 – APC-Cy7 | 1 | ICS-tmr | Biolegend | 512320 |
| CD69 – BV785 | 2.5 | ICS-tmr | Biolegend | 310931 |
| CD4 – BV510 | 1 | ICS-tmr | BD | 562970 |
| CD45RA – PE-Cy7 | 1.5 | ICS-tmr | Biolegend | 304126 |
| CD4 – APC | 2.5 | In vitro tmr | BD | 555349 |
