# Supplementary material for: Efficient ex vivo analysis of CD4+ T-cell responses using combinatorial HLA class II tetramer staining
Source: Nat Commun. 2016 Aug 30;7:12614. doi: 10.1038/ncomms12614 (PMC5013714; doi:10.1038/ncomms12614)
Supplement: Peer review file [file ncomms12614-s2.pdf]

Reviewers' Comments:

Reviewer #1 (Remarks to the Author)

Review of "Efficient ex vivo analysis of T-cell responses using combinatorial HLA-class II tetramer staining" by Hannes Uchtenhagen et al.

In this manuscript, the authors have carefully optimized and validated the application of combinatorial peptide-MHC tetramer staining for using with HLA-DR tetramers. This validation was thorough and the quality of the data is very high. To demonstrate the utility of this approach, they have analyzed the frequencies, phenotypes and functional profiles of influenza specific cells in a small cohort of donors evaluated before and after seasonal influenza vaccination. In addition to mapping novel influenza T cell epitopes, the authors report a number of interesting observations about the antigen-specific T cell response in the context of vaccination. They also explore T cell crossreactivity for two epitopes with similar sequences.
Although the novelty of the approach is incremental from published studies using MHC class I tetramers, this work is important and should facilitate future studies involving the use of MHC class II tetramers. This is a carefully performed study with high quality data, figures and writing.

Major comment:

The authors were careful to explain that the combinatorial tetramer staining approach only permits the identification of uniquely specific T cells and this is important. I think it would be important to expand on this discussion and the extent to which crossreactivity would affect the interpretation of all figures that employed the combinatorial staining strategy. The very good correlation between single staining and combinatorial staining show in supplementary Fig. 4 is very nice, but can any of these small deviations be explained by crossreactivity? It would also be useful for the authors to comment about how this factor should be considered in future studies. Do they agree that this issue would come into play mostly in cases when the sequences of epitopes are similar as is the case for the TxHA-321 and PeHA-322 epitopes? Did authors investigate crossreactivity for any other epitope combinations?

Minor comments:
Would it be possible to back-calculate pre-enrichment antigen-specific cell frequencies in Fig. 1? This would make it easier to assess the performance of each of the fluorophores and the comparison of single vs. combinatorial staining in panel c.

What was the basis for deciding which fluorophore was suboptimal?

Did the authors investigate the quantum dots for MHC class II multimer staining? How do these compare with the other dyes used here?

Fig. 2b it is difficult to see each of the points with the extent of jitter chosen? Although it might be difficult to fit nicely in this figure, it would be nice if dots could be connected for each donor to show donor-to-donor variation in vaccine response. Maybe it is not necessary to show the pre-vaccination frequencies twice - once alone in panel a. and again together with post vaccination in panel b? Instead maybe a larger figure could be created that makes it easier to see all points, and if possible connects the points for each donor tested.

I think it would be nice to specify in the figure legends which figures are showing data generated using multiplex tetramer staining vs. those generated using single fluorophores.

Reviewer #2 (Remarks to the Author)

In the manuscript by James and colleagues, the authors use multiple MHC Class II tetramers of influenza epitopes to optimize detection of virus-specific CD4 T cells in human blood prior to and following vaccination. They analyze blood obtained from 6 individuals between the ages of 42-67 prior to and after influenza vaccination, and quantitate flu-specific CD4 T cells based on different epitopes. Analysis of antigen-specific CD4 T cells in humans is challenging and often requires tetramer enrichment, necessitating large numbers of cells. Here, the authors added a c-myc tag to the HLA molecule which they used to make MHC Class II tetramers, enabling enrichment with an anti-myc antibody. This approach enabled increased sensitivity for detecting epitope-specific T cells. The main results of this study demonstrate excellent sensitivity of this technique. However, they don't show any new results either concerning the nature or phenotype of flu-specific CD4 T cells in human blood, and the sample size of 6 for analysis of epitope-specific T cell frequency before and after vaccination is too small to make significant conclusions. Moreover, the 6 samples analyzed are shown as bulk and the links between individuals before and after vaccination are not shown so we don't know if an individual showed an increase or no change in the frequency of epitope-specific T cells. Similarly, the analysis of T cell cross-reactivity only shows results from three samples and is also too small for significant comparisons. This study is more of a technical note, rather than a full testing of their technique to show some new insight or result on peripheral blood CD4 T cell responses.

Reviewers' comments:

Reviewer #1 (Remarks to the Author):

Review of "Efficient ex vivo analysis of T-cell responses using combinatorial HLA-class II tetramer staining" by Hannes Uchtenhagen et al.

In this manuscript, the authors have carefully optimized and validated the application of combinatorial peptide-MHC tetramer staining for using with HLA-DR tetramers. This validation was thorough and the quality of the data is very high. To demonstrate the utility of this approach, they have analyzed the frequencies, phenotypes and functional profiles of influenza specific cells in a small cohort of donors evaluated before and after seasonal influenza vaccination. In addition to mapping novel influenza T cell epitopes, the authors report a number of interesting observations about the antigen-specific T cell response in the context of vaccination. They also explore T cell crossreactivity for two epitopes with similar sequences.
Although the novelty of the approach is incremental from published studies using MHC class I tetramers, this work is important and should facilitate future studies involving the use of MHC class II tetramers. This is a carefully performed study with high quality data, figures and writing.

We appreciate the positive feedback regarding the technical aspects of our work and are glad that this reviewer considers our observations to be of interest.

Major comment:

The authors were careful to explain that the combinatorial tetramer staining approach only permits the identification of uniquely specific T cells and this is important. I think it would be important to expand on this discussion and the extent to which crossreactivity would affect the interpretation of all figures that employed the combinatorial staining strategy. The very good correlation between single staining and combinatorial staining show in supplementary Fig. 4 is very nice, but can any of these small deviations be explained by crossreactivity? It would also be useful for the authors to comment about how this factor should be considered in future studies. Do they agree that this issue would come into play mostly in cases when the sequences of epitopes are similar as is the case for the TxHA-321 and PeHA-322 epitopes? Did authors investigate crossreactivity for any other epitope combinations?

We thank this reviewer for the astute suggestion that T cell cross reactivity might occur not only for structurally similar epitopes (such as TxHA-321 and PeHA-322) but also for structurally dissimilar epitopes. In this revision we utilize a more comprehensive strategy for assessing cross-reactivity present in our combinatorial tetramer data by broadly evaluating the number of cells that were labeled by 3 or 4 different colors of tetramers. This allowed us to estimate the degree of cross-staining for different pairs of epitopes (now shown in Figure 5a and Supplementary Figure 9). In this way, we were able to clearly demonstrate that the TxHA-321 peptide had a significantly higher proportion of cells that showed evidence of cross reactivity than other non-homologous pairs of epitopes. Therefore, for the epitopes that were included in our study, significant cross-reactivity was only observed for structurally similar epitopes. Admittedly, our approach is inherently limited by the fact that low levels

of cross-reactivity cannot be readily distinguished from background staining, which accounts for the presence of low numbers of 3 and 4 color tetramer positive events that were seen across the five pairs of non-homologous peptides.

Beyond this, we now discuss in more detail the implications that removing the cross-reactive (3 and 4 color tetramer positive events) events could have for the different figures, including Supplementary Fig. 4. Given the low level of cross reactivity for the other pairs of epitopes, the overall impact on most of our findings is minimal.

We agree that the topic of cross-reactivity within the TCR repertoire could have wider significance for antigen specific T cell research. In scenarios where large numbers of epitopes are visualized in parallel, unexpectedly high levels of cross-reactivity between different epitopes could be observed de novo using the approach that we have outlined here. Conceptually, observing a broad tolerance for a given TCR to changes in its MHC/peptide binding interface could be of particular importance for the development of broadly protective vaccines or could be a distinguishing feature of pathogenic autoreactive T cells. Relevant comments about this topic have been added to the discussion.

Minor comments:
Would it be possible to back-calculate pre-enrichment antigen-specific cell frequencies in Fig. 1? This would make it easier to assess the performance of each of the fluorophores and the comparison of single vs. combinatorial staining in panel c.

Theoretically it can be possible to determine T cell frequencies using a non-enriched tube – if the T cell frequency is high enough and if an adequate number of non-enriched cells is analyzed. Unfortunately, the experimental design of our study dictated that we reserve only 1% of the total sample for pre-enrichment analysis (preserving as many cells as possible for post-enrichment analysis). As a result, we found that we were not able to calculate meaningful frequencies from the non-enriched sample.

Conceptually, observed differences in the performance of tetramers conjugated with different fluorophore labeled streptavidin for ex vivo tetramer analysis could originate from differences in the efficacy of enrichment or due to differences in labeling (or both). Our data suggest that the observed differences were apparently not caused by the method of enrichment, as either anti-PE or anti-Myc enrichment of PE-labeled cells gave essentially identical results (Supplementary Fig. 1). We had previously observed for tetramer labeling of in vitro expanded T cells (Yang et al Clin Immunol 2006), that conjugation of class II tetramers from streptavidin labeled with different fluorophores results in reagents that label T cells with different efficacies. This would suggest that differences in the efficacy of labeling can occur independent of any enrichment step. Based on our experience, we believe that tetramers labeled with large fluorophores such as PE have more favorable performance in terms of staining efficacy because they exhibit an increased tendency to aggregate into higher order multimers, facilitating improved labeling – especially of lower affinity cells. Corresponding comments have been added to the results and discussion.

What was the basis for deciding which fluorophore was suboptimal?

Pragmatically, we were looking to identify various labels that would provide a consistent readout of epitope specific T cell frequency and that could be used together without creating compensation issues. Primarily we made our evaluation based on the observed T cell frequency, using PE labeled tetramers as a benchmark. Our secondary consideration was the MFI of tetramer staining as compared with background staining. In this revision we have added additional comments to clarify or rationale for selecting the fluorophores that were included in our final panel.

It is possible that other MHC multimer backbones (such as dextramers or dodecamers) could enable the use of additional fluorophores that have not performed well in our studies. For example, there has been some success staining CD4+ T cells with FITC labeled dextramers (Massilamany et al BMC Immunology 2011). Comments about this have been added to the discussion

Did the authors investigate the quantum dots for MHC class II multimer staining? How do these compare with the other dyes used here?

We have tried Qdot multimers with some promising but mixed results. In particular, we had issues with a lack of staining consistency. We and others (David Price of Cardiff University, personal communication) have noted that conjugated Qdot multimers have a relatively short shelf life, which may explain these problems.

Fig. 2b it is difficult to see each of the points with the extent of jitter chosen? Although it might be difficult to fit nicely in this figure, it would be nice if dots could be connected for each donor to show donor-to-donor variation in vaccine response. Maybe it is not necessary to show the pre-vaccination frequencies twice - once alone in panel a. and again together with post vaccination in panel b? Instead maybe a larger figure could be created that makes it easier to see all points, and if possible connects the points for each donor tested.

As requested, we have deleted the original Figure 2a) with the Day 0 frequencies and have reformatted the figure to increase the visibility of the vaccine response data. The graph also has been modified to show matched before-after responses as requested by reviewer #2.

I think it would be nice to specify in the figure legends which figures are showing data generated using multiplex tetramer staining vs. those generated using single fluorophores.

This is now included in each of the figure legends as requested

Reviewer #2 (Remarks to the Author):

In the manuscript by James and colleagues, the authors use multiple MHC Class II tetramers of influenza epitopes to optimize detection of virus-specific CD4 T cells in human blood prior to and following vaccination. They analyze blood obtained from 6 individuals between the ages of 42-67 prior to and after influenza vaccination, and quantitate flu-specific CD4 T cells based on different epitopes. Analysis of antigen-specific CD4 T cells in humans is challenging and often requires tetramer enrichment, necessitating large numbers of cells. Here, the authors added a c-myc tag to the HLA molecule which

they used to make MHC Class II tetramers, enabling enrichment with an anti-myc antibody. This approach enabled increased sensitivity for detecting epitope-specific T cells. The main results of this study demonstrate excellent sensitivity of this technique.

We appreciate the positive feedback regarding the technical aspects of our work. We agree that our approach provides an efficient and sensitive means for characterizing antigen specific CD4+ T cell responses in a variety of settings

However, they don't show any new results either concerning the nature or phenotype of flu-specific CD4 T cells in human blood

The reviewer's point regarding the lack of novelty in our results is well taken. In this revision we have analyzed two additional samples sets to increase the depth and novelty of our analysis of influenza specific CD4 T-cells in human subjects:

1) We directly visualized influenza specific CD4 T-cells in human cord blood samples, allowing us to draw inferences about the epitope hierarchy of naïve CD4 T-cells and the influence that this hierarchy might have on responses to novel influenza strains

2) We characterized influenza specific CD4 T-cells in rheumatoid arthritis patients before and after vaccination to investigate the possible effects of immune modifying therapies on vaccine responses.

The specific scientific insights from these new experiments significantly extend the scope of our work beyond the original manuscript and underscore the usefulness of the developed protocol to analyze CD4 responses from limited clinical samples in various settings.

The sample size of 6 for analysis of epitope-specific T cell frequency before and after vaccination is too small to make significant conclusions.

Because of the time-sensitive nature of studying responses to vaccination, we were unable to add more subjects to the experiments shown in fig 2 and 3 or extend the analysis through the inclusion of additional time-points after vaccination (additional samples are not available in our sample repository and no one is receiving that vaccine at present) .

We acknowledge that larger subject groups would have been desirable and agree that the limited number of subjects leaves our study underpowered to detect subtle but interesting phenotypic differences that may be present in the influenza specific T cells of vaccine recipients. However, we would emphasize that the distribution of our frequency data was quite tight and that changes in frequency were consistent among the subjects tested. Despite the small number of subjects, conventional statistical analysis supports the significance of our findings with respect to T cell frequency. Therefore we would contend that, despite its small size, our study allowed us to draw conclusions influenza specific T cells that are valid and of interest. Out of deference to this concern, we have added comments to the discussion about the limitations and caveats caused by the small size of our study.

Moreover, the 6 samples analyzed are shown as bulk and the links between individuals before and after vaccination are not shown so we don't know if an individual showed an increase or no change in the frequency of epitope-specific T cells.

The reviewer is correct that changes in T cell frequency before and after vaccination were obscured in our original "bulk" presentation of the data. In this revision we have modified figure 2a as requested to show matched before-after responses for each individual. As presented, we believe that it is now clear that changes in T cell frequency pre and post-vaccination were, in general, quite consistent among these different subjects.

Similarly, the analysis of T cell cross-reactivity only shows results from three samples and is also too small for significant comparisons.

As requested we have extended our cross-reactivity experiments to include all six subjects. As summarized in our detailed response to reviewer #1, we also extended that analysis to draw inferences about the degree of cross-reactivity between structurally similar epitopes (TxHA-321 and PeHA-322) versus structurally dissimilar epitopes. We believe that our data now makes a statistically valid case for the specific cross-reactivity between TxHA-321 and PeHA-322, thereby illustrating the utility of the combined combinatorial staining approach for the investigation of CD4 cross-reactivity amongst pairs of homologous and non-homologous epitopes.

This study is more of a technical note, rather than a full testing of their technique to show some new insight or result on peripheral blood CD4 T cell responses.

Through the addition of additional data from human cord blood samples and rheumatoid arthritis patients who received the seasonal influenza vaccine, we have significantly extended the scope of our work beyond the original manuscript. In our opinion, the combination of our original findings and this new data constitutes a more adequate breadth and depth of application for our technique and provides scientific insights that would be of interest to the readers of this journal. We hope that this reviewer concurs with our assessment.

Reviewers' Comments:

Reviewer #1 (Remarks to the Author)

My comments have been adequately addressed.

Reviewer #2 (Remarks to the Author)

The authors have added some additional analyses and data to this study, including the frequency of influenza-epitope-specific T cells prior to and following vaccination and data from cord blood and from a small cohort of patients with rheumatoid arthritis. However, the findings, in my opinion still represent more of a technical advance in detection of low frequency populations of antigen-specific T cells in human blood and I do not perceive any novel or new insights on the influenza-specific T cells in humans either during steady state or following vaccination. The variability in inducing increased frequencies of flu-specific T cells before and after vaccination is confirmed in this study, as well as the preponderance of memory T cells, and the study itself is not sufficiently powered for any statistically relevant conclusions to be drawn. The very low frequency of influenza-specific T cells in naïve cord blood samples is also known and doesn't add to the analysis. The inclusion of vaccine studies from a cohort of 6 patients with rheumatoid arthritis being treated with different biologics is not sufficiently powered for any real conclusions to be made and doesn't really belong with the study. There is no autoimmunity control or even healthy controls done at the same time and it is not clear why this part was added. The manuscript also lacks the statistical methods part.

Reviewers' comments:

Reviewer #1 (Remarks to the Author):

My comments have been adequately addressed.

Reviewer #2 (Remarks to the Author):

The authors have added some additional analyses and data to this study, including the frequency of influenza-epitope-specific T cells prior to and following vaccination and data from cord blood and from a small cohort of patients with rheumatoid arthritis. However, the findings, in my opinion still represent more of a technical advance in detection of low frequency populations of antigen-specific T cells in human blood and I do not perceive any novel or new insights on the influenza-specific T cells in humans either during steady state or following vaccination. The variability in inducing increased frequencies of flu-specific T cells before and after vaccination is confirmed in this study, as well as the preponderance of memory T cells, and the study itself is not sufficiently powered for any statistically relevant conclusions to be drawn. The very low frequency of influenza-specific T cells in naïve cord blood samples is also known and doesn't add to the analysis. The inclusion of vaccine studies from a cohort of 6 patients with rheumatoid arthritis being treated with different biologics is not sufficiently powered for any real conclusions to be made and doesn't really belong with the study. There is no autoimmunity control or even healthy controls done at the same time and it is not clear why this part was added. The manuscript also lacks the statistical methods part.

We agree that our study would be greatly improved through a more consistent and rigorous statistical analysis of our data. As outlined in our response to the biostatistics expert below, we now employ a more unified statistical analysis, including parallel application of parametric and non-parametric tests and a more careful statistical treatment throughout. With this analysis it is clear that, despite the small sample size of our study, our key findings remain significant even under the full rigor of non-parametric testing with multiple testing correction. We would contend that several of our findings about epitope cross reactivity and about the epitope hierarchy of naïve CD4 T-cells and the influence that this hierarchy might have on responses to novel influenza strains reflect novel findings that will be of interest to the readers of Nature Communications.

Biostatistician (Remarks to the Author):

There are a few statistical issues that need to be addressed.

1. The authors were inconsistent across the in what type of statistical test they were using for the comparisons in Figure 2, sometimes a paired Wilcoxon (non-parametric), sometimes an ANOVA (parametric). The same comment applies throughout, in that pvalues in other figures and supplemental figures are sometimes parametric and sometimes non-parametric. There is no apparent reason to have done this given the sample size is the same, and there are non-parametric choices for all tests done. This reviewer wonders if the ANOVA test chosen for Figure 2 was the one that would provided stronger statistical significance, compared to a non-parametric test- which is not a valid motivation for the

choice. The choice of parametric or non-parametric seems better to be consistent across the analysis -or better discussion of why it is switching around. If the authors choose to use parametric tests, the limitations of using a parametric test with such small samples needs to be acknowledged, say in discussion of the paper, particularly since there is some apparent skewness in some of the data. Reporting both the parametric and non-parametric p-values in all cases is another approach that could be considered, certainly preferred over the mixed approach currently taken.

In response to this critique we have added a statistical methods section to the paper that delineates the specifics of our analysis. We are now providing both parametric and non-parametric p-values, having performed such tests for the data presented in all of our figures. We have provided a rationale for the specific tests chosen in the Statistics paragraph - together with a statement regarding the possibility that not all distributions are normal, thereby highlighting the limitations of parametric tests in the instance where normality of the data is in question due to small sample size. This provides the reader with the opportunity to understand which of the significant findings are robust and which are more preliminary. The tetramer frequency data displayed in Fig. 2a, 4 and Supplementary Fig. 10 is displayed on a log axis and appears to be non-normally distributed on a linear scale. Therefore, it has been log-transformed before performing statistical tests.

2. The legend says that the Bonferroni test for multiple comparisons were used for pvalues in Figures 2b and 2c, but it is unclear, whether the adjusted p displayed in the graph, or the raw p. This should be made more clear, it would also be good to say what level you were using for significance in the Bonferroni test - in order to make clearer exactly how many comparisons were counted and corrected for.

In this revision, all of the displayed p values are shown after correction for multiple testing whenever applicable and this is now clearly stated in the methods section. A p value of 0.05 (after correction whenever applicable) has been chosen as cut-off for significance and, correspondingly, p-values equal to or greater than 0.05 are shown in parenthesis.

Was it all pairwise comparisons? How did you decide which comparisons to adjust for - for example in Figure 3c did you only Bonferroni adjust the comparisons that looked significant, so subjects 2-6 versus subject 1 only. The latter is not a reasonable approach for multiple comparison adjustment. This comment applies to the other instances where Bonferroni adjustment is made: eg. Figure 3,5, Supp Figure 6

We have now made a clearer and consistent articulation of our choice of test and the pairwise comparisons employed both in the figure legends and methods paragraph. In Figure 3a, 3d, 5a, 5d all possible pairwise comparisons have been performed and p values corrected accordingly.

Furthermore, we have removed the statistical analysis of Figure 3b as it is a preliminary finding that is not an important point in the manuscript and at least to some extent driven by what appears to be one biological outlier. We therefore make no claims that this finding is significant.

In Figure 4 and Supplementary Figure 6 only, selected pairwise post tests have been performed rather than all possible tests. This was not based on whatever appeared significant but rather followed the specific hypotheses of these experiments (that was formulated in advance). In Figure 4 we set out to test whether the two conserved but subdominant epitopes are indeed lower than a newly introduced and a dominant conserved epitope in adults and cord blood. In Supplementary Figure 6a we set out to test whether the memory frequencies of the two newly introduced epitopes are lower than the rest. In Supplementary Figure 6b we set out to test whether the CD38 frequencies of two epitopes that failed to upregulate (before versus after) vaccination are lower than that of the other epitopes. The relevant columns have been highlighted in each Figure and the fact that limited comparisons were made is now evident.

3. Supp Table 6 legend seems to be describing the two graphs in part (a) only (top left and top right), in that the text listed for part (b) seems to be relevant the graph to the right for part a. Also a typo, listing $p>0.001$, when graph says $p <0.001$.

We appreciate the comment and have corrected the figure legend accordingly.
